# Effects of progressive body-weight versus barbell back squat training on strength, hypertrophy and body fat among sedentary young women

**DOI:** 10.1038/s41598-023-40319-x

**Published:** 2023-08-19

**Authors:** Wei Wei, JingX Zhu, Shuang Ren, Yih-Kuen Jan, WuL Zhang, Ronghai Su, Li He

**Affiliations:** 1 Sports and Health Improvement Research Center of College of Physical Education and Sports, Beijing Normal University, Xinjiekouwai Street 19, Haidian District, Beijing, 100875 China; 2 The Sports Medicine Laboratory of Peking University Third Hospital, Peking University Health Science Center, Beijing, China; 3 Department of Kinesiology and Community Health, University of Illinois at Urbana-Champaign, Champaign, USA

**Keywords:** Public health, Musculoskeletal system

## Abstract

The objective of this study was to compare the effects of progressive bodyweight training and barbell back squat on muscle strength, muscluar hypertrophy, and body fat percentage in sedentary young women. Thirteen sedentary young women (aged 19.77 ± 0.83 years, height 164.91 ± 6.01) were randomly assigned to either the progressive bodyweight group (n = 6, consisting of 10 levels of movements progressing from bilateral to unilateral) or the barbell squat group (n = 7, 60–80% 1RM). Both groups underwent two training sessions per week for 6 weeks. Measurements of muscle strength (isokinetic knee extensor and flexor muscle peak torque of each leg), muscle thickness (gluteus maximus, rectus femoris, and gastrocnemius muscles), and body fat percentage were taken at baseline and post-testing. Both groups showed a significant increase in isometric peak torque of the knee extensor and flexor (p < 0.05), but there were no significant between-group differences in isometric peak torque of the knee extensor and flexor (p > 0.05) or in the mean concentric peak torque of the knee H/Q ratio (p > 0.05). Both groups also showed significant increases in muscle thickness (p < 0.05), with no significant differences in Gastrocnemius, Rectus femoris and Gluteus maximus (p > 0.05). The percentage of body fat significantly decreased in the barbell group (pre: 28.66 ± 4.58% vs post: 24.96 ± 5.91%, p = 0.044), but not in the bodyweight group (pre: 24.18 ± 4.63% vs post: 24.02 ± 4.48%, p = 0.679). Our findings indicate that while both training methods increased maximum strength and muscle mass, barbell back squat training may be more effective in reducing body fat percentage.

## Introduction

Sedentary behavior has become increasingly common due to lifestyle changes. Long time of sedentary behavior not only can lead to high risks of chronic diseases such as cardiovascular disease, diabetes, and cancer^1^, but also lead to increased pressure on intervertebral discs, lumbar stiffness, and lower back pain^2–4^, as well as knee pain and osteoarthritis^5^. Particularly, sedentary women are at a higher risk of knee injury during sports than men^6^, which makes leg strength is crucial for knee joint protection and preventing muscle mass and bone loss in the lower limbs among them^7,8^.

The squat exercise is a kind of multiple-joint exercise mainly to increase the muscle strength and muscular hypertrophy of lower body muscles that descent and ascent the body center of gravity by flexing and extending the hip, knee and ankle^9^. One kind of the popular squat exercise—barbell squat—has been widely used to increase lower body muscle strength and muscular hypertrophy by flexing and extending the hip, knee, and ankle^9^. It can control training intensity according to the individual’s 1RM (One repetition maximum) through adjust the weight of the barbell plate, so as to gradually increase the load volume (load volume = load intensity × repetitions × sets) with the progressive improvement of the strength and muscular hypertrophy^10–12^. Existing studies advice that increasing the maximum strength and hypertrophy of muscle through barbell squats should be controlled the load volume at 3–6RM (93–85%1RM) and 6–12RM (85–70%1RM)^13^, while some studies suggest that 3–8RM (93–80%1RM) and 8–15RM (80–60%)^14^. However, studies have shown that novice women may struggle with proper posture during deeper barbell squat movements^15,16^, and barbell back squats may increase the risk of knee and lumbar intervertebral disk injuries^17,18^. These findings implied that, for women who have been sedentary for a long time, barbell squats may not be the best exercise for reducing lumbar load and injury risk.

On the other hand, bodyweight squats, which promote lower limb muscle strength by working against the gravity of the body's own weight, have also been widely used by increasing repetitions, shortening the rest-interval time, changing the squat angle, or increasing the difficulty of movement under constant load (weight)^19–22^. Increasing repetitions or shortening rest intervals may lead to greater endurance improvement rather than maximum strength^20–22^. In this respect, researchers proposed that squat training using a variety of postures to overcome body weight can improve not only muscle strength, but also muscle coordination, joint stability, and functional training, such as balance and flexibility, as opposed to different weights of barbell in a stationary position or repetitions under constant bodyweight squat posture^23^. For example, Begalle et al.^24^ found that unilateral bodyweight squats can improve quadriceps-to-hamstrings coactivation ratio, promoting knee stability and injury prevention. Knoll et al.^25^ found different muscle activation between traditional Split Squats and single leg squat variation using EMG, where the traditional Split Squats had more activation of quadriceps muscles, while single leg squat variation (single leg forward leaning squat) had more activation of gluteus maximus. Accordingly, different effects on muscle activation of different squat postures has been proved, so that changing postures can be a way to progress training load^25–27^. However, although the transition from bilateral to unilateral body weight squats in various combinations of positions and angles has demonstrated acute effects on the lower extremities muscle, 74.5% of adults in the U.S. do not meet physical activity guideline recommendations^28^, and the estimated participation is even lower when specific activities such as RT are considered^29^. Therefore, it is very important to the relationship between exercise effect and cost and whether it can effectively promote the lower limb strength and muscle hypertrophy level of the exerciser. Compared with the barbell squat, which can clarify the load volume, it is necessary to determine the effect of bodyweight squat by gradually changing the posture and joint Angle to improve the lower limb muscle strength and muscle hypertrophy. On the one hand, in order to provide a suitable set of progressive bodyweight squat training methods for the general population, it is necessary to determine its long-term training effect on muscle strength and muscle hypertrophy. On the other hand, the bodyweight squat is not limited by the site and equipment, which may makes it more cost-effective and better to promote public participation in resistance training.

The present study aimed to compare the effects of 6-week progressive barbell-back and bodyweight squat training programs on muscle strength, thickness, and body composition in sedentary young women. This will provide scientific evidence for designment of effective training programs for females. We hypothesized that bodyweight squats would significantly improve knee joint strength, lower limb muscle hypertrophy, and body fat and that barbell-back squats and barbell squats would have similar effects on strength and muscle hypertrophy.

## Method

### Study design

The study utilized a parallel, single-blind, randomized controlled trial design (RCT). To divide the participants into two groups, a simple randomization system was employed, namely the in-college course selection system, which required the participants to choose one of the two groups. The participants were given the option to randomly and voluntarily join either group via the system. 13 participants were selected from two supervised programs and followed the same progressive resistance training principles for the entire 8-week period. Each program consisted of two weekly training sessions for eight weeks, with two weeks allocated for familiarization and six weeks for formal intervention.

### Subjects

**Figure 1:**
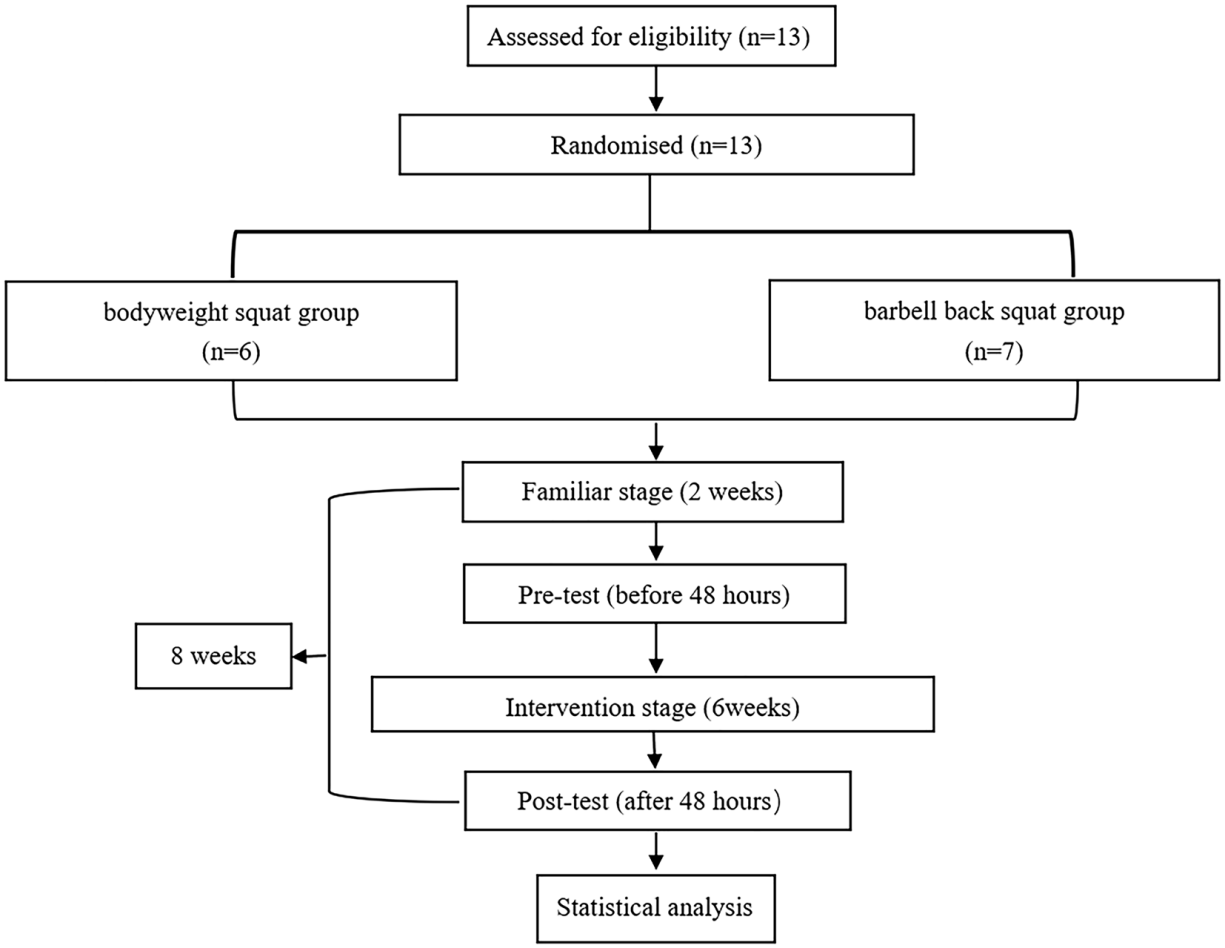
Flow chart of the present study.

Thirteen young women, with an average age of 19.77 ± 0.83 years, were conveniently recruited from Beijing Normal University for this study. The participants were required to meet specific criteria, including being between 18 and 30 years old, weight stable (with a weight change of < 3% of body weight) for at least 6 months (with a BMI < 30), inactive (< 150-min of moderate to vigorous physical activity per week), not taking any nutritional supplements, and free of medical problems that could be excluded by the study protocol. A validated medical screening questionnaire was used to screen for physical or mental health problems such as cardiovascular disease, patellar injury, muscle injuries, orthopedic problems, motion-limiting osteoarthritis, fibromyalgia, and depression. Thirty-six sedentary young women were recruited, ultimately, thirteen sedentary young women were able to complete the study (Fig. 1).

The study protocol and experimental procedures were explained to each participant, who then provided informed consent. The testing and training sessions took place at the College of P.E. and Sports at the Beijing Normal University and the Institute of Sports Medicine at the Third Hospital of Peking University, and followed the ethical guidelines of the Declaration of Helsinki for the study of humans. The Experiment of Sports and Health Promotion Research Center, College of P.E and Sports, Beijing Normal University of Ethics Committee reviewed and approved the studies involving human participants. Written informed consent was obtained from all participants to participate in the study, and to publish any potentially identifiable images or data included in the article.

### Procedures

Each session lasted 60 min and was separated by at least 48 h. The sessions comprised of a 15-min warm-up consisting of 10 activities, followed by a 30-min activity segment of squat exercises, with each group performing 6 sets of either bodyweight or barbell squats. Finally, there was a 15-min cool-down consisting of 8 activities. The warm-up and cool-down exercises were the same for both experimental groups. All sixteen training sessions were supervised by two experienced exercise instructors, who paid special attention to the consistency of the movement pace^30^. The instructors were physical education and training undergraduates with at least 3 years of resistance training experience. They controlled the velocity of the concentric and eccentric phases during each squat for 2 s through verbal cadence in each session^31^. Additionally, two assistants recorded videos of each session, and after training, one of the assistants evaluated the quality of squatting movement using a self-developed movement quality scale while watching the videos (Attached supplementary S2).

### Familiarization session

During the first 2 weeks (Weeks 1–2), the participants attended four familiarization sessions at the gym to get acquainted with the exercise equipment and protocol in their respective groups, including key points of attention in each activity.

In the first week, the participants in the barbell group completed six sets of ten repetitions of barbell squats without disks, while the participants in the bodyweight squat group followed the progressive bodyweight training protocol and performed six sets of bodyweight squats. In the second week, the participants were required to attend baseline assessments twice, with intervals of 48 h based on their groups, to determine the starting level of bodyweight squat movement in the bodyweight group and the one-repetition maximum of the barbell squat in the barbell squat group. After the baseline assessments, the lowest values (one repetition maximum and initial squat level) of participants in the two groups were used as the evaluation results to determine the load in the formal intervention. The evaluation approach of bodyweight squat movement level is based on the progressive push-up level assessment used in the research by Kotarsky et al.^32^.

Forty-eight hours after the 2 weeks of familiarization visits, another baseline assessment of height, weight, body fat, and muscle strength and thickness were conducted in the laboratory of the Beijing Normal University and the Third Hospital at Peking University. The evidence strongly suggests that the 2-week short-term training would not have a significant impact on the physiological indicators of subjects^33^.

From week 3 (weeks 3–8), all participants started the formal intervention in each experimental condition. The intervention included 60-min sessions separated by at least 48 h, comprising of 15-min warm-up activities (10 activities, 15-min), 30-min squat exercises (6 sets of bodyweight or barbell-squat for each group), and 15-min cool-down exercises (8 activities, 15-min). Both the warming and cool-down exercises were the same in the two experimental groups. All 16 training sessions were supervised by two experienced exercise instructors, and special attention was given to the consistency of the movement pace^30^.

### The progressive bodyweight squat program

The progressive bodyweight squat protocol consisted of 10 levels of squats from A to J, as described in supplementary S1^34^. Each participant’s squat level was gradually increased over the 6-week period based on the following principles. During each training session, all participants performed 6 sets of squats, including 4 sets at the initial level and 2 sets of squats at the two sequentially lower levels. The number of repetitions decreased with each set, and the specific number was based on the quality of movement and intensity of each participant's performance. The rest period of each set was 2 min, similar to the barbell back squat group. The intensity of each session was measured by the Ratings of Perceived Exertion (OMNI-Resistance Exercise Scale), which was completed independently by each participant after each training^35^. The total volume of repetitions performed in the 12 training sessions (6 weeks) was calculated.

During the familiarization sessions, all participants began with level C for the first set. If they could correctly complete 10 repetitions for double leg squat on four sets at level C in two consecutive training sessions, they could advance to level D. For single leg squat, participants had to perform 5 repetitions per side for single leg squat on four sets at level F in two consecutive training sessions to advance to level G. For example, participants who began with level C were required to perform six sets of bodyweight squats, including 12 repetitions for double leg squats at level A, 10 repetitions for double leg squats at level B, and four sets of double leg squats at level C (8 repetitions per set). In total, during an exercise session, they were required to complete the following: 12 (A)/10 (B)/8 (C)/8 (C)/8 (C)/8 (C)/8 (C). From the first training session in the formal intervention (week 3), participants gradually increased to 12 (E)/10 (F)/8 (G)/8 (G)/8 (G)/8 (G) after achieving 12 repetitions of level E, 10 repetitions of level F, and 4 sets of 8 repetitions of level G in that session (4 repetitions per side). In the next session, they aimed to finish 12 (E)/10 (F)/10 (G)/10 (G)/10 (G)/10 (G) (5 repetitions per side). If participants could perform 12 (E)/10 (F)/10 (G)/10 (G)/10 (G)/10 (G) in two consecutive sessions, they could progress to level H. Participants who began with 12 (F)/10 (G)/8 (H)/8 (H)/8 (H)/8 (H) followed the same pattern to progress throughout the 6-week training. When participants could not complete 8 repetitions for double leg squats or 4 repetitions on each side for single leg squats at each level, the progression ended, and they began again at the previous level. An example is shown in Table 1. The bodyweight squat progression protocols were shown in the supplementary S3.

**Table 1:** Bodyweight squat progression.

Subjects	First session		Second session		Third session		Fourth session	
	Level	R × S	Level	R × S	Level	R × S	Level	R × S
1	E/F/G/G/G/G	12/10/8/8/8/8	E/F/G/G/G/G	12/10/10/10/10/8	E/F/G/G/G/G	12/10/10/10/10/10	E/F/G/G/G/G	12/10/10/10/10/10
2	E/F/G/G/G/G	12/10/8/8/8/8	E/F/G/G/G/G	12/10/10/10/8/8	E/F/G/G/G/G	12/10/10/10//10	E/F/G/G/G/G	12/10/10/10/10/10
3	E/F/G/G/G/G	12/10/8/8/8/8	E/F/G/G/G/G	12/10/10/10/10/8	E/F/G/G/G/G	12/10/10/10/10/10	E/F/G/G/G/G	12/10/10/10/10/10
4	E/F/G/G/G/G	12/10/8/8/8/8	E/F/G/G/G/G	12/10/10/10/10/8	E/F/G/G/G/G	12/10/10/10/10/10	E/F/G/G/G/G	12/10/10/10/10/10
5	E/F/G/G/G/G	12/10/8/8/8/8	E/F/G/G/G/G	12/10/10/10/10/8	E/F/G/G/G/G	12/10/10/10/10/10	E/F/G/G/G/G	12/10/10/10/10/10
6	E/F/G/G/G/G	12/10/8/8/8/8	E/F/G/G/G/G	12/10/10/8/8/8	E/F/G/G/G/G	12/10/10/10/10/10	E/F/G/G/G/G	12/10/10/10/10/10

Note: Repetitions × Sets = R × S; 1–6 represents each participant; E: Lunge, F: Bulgarian single leg squat, G: Skating squat; the table shows the training volume and movement level (A–J) completed by the 6 participants in the bodyweight squat group in the first four training sessions;

### The progressive barbell-back squat program

During the familiarization session, participants performed exercises under the guidance of two trained instructors. All subjects attended two training sessions in the first week to get familiarized with the equipment and squat techniques, and to ensure they understood the proper form. The instructors used a barbell (20 kg) and weight plates with different loads to measure each participant's one-repetition maximum (1RM) in the second week. Using a multiple-repetitions testing procedure, participants were required to perform 5 sets of 5 repetitions of their maximum load of squats to estimate their 1RM. A jump box was placed behind participants to ensure proper squat form, with their hips and thighs close to the box and their thighs parallel to the ground, while their knees did not extend beyond their toes. The barbell squat movement adopted a high-bar back squat action ^4^(12). Participants were instructed to descend until their thighs were parallel to the ground, with the greater trochanter of the femur forming a horizontal line with the upper end of the patella, and the thigh and hip parallel to the box and ground as the visual cue. They were then instructed to rise to the starting position after receiving verbal guidance from the instructors.

Before the test, participants had their barbell-back squat technique critiqued and corrected during the first and second familiar sessions in the first week, and became familiar with the equipment. The test was preceded by a warm-up set of 8 repetitions of barbell squats without plates. Participants then performed sets at progressively increasing loads until they failed to complete a valid repetition, judged by their inability to complete the full range of motion. Ideally, subjects failed within 3–5 repetitions during the last and heaviest set^12^. From the second set, each set involved 5 repetitions of squats and progressed by the addition of two plates (2.5 kg per plate) in the subsequent set until participants were unable to complete a set. There was a 2-min break between sets. The 1RM was calculated according to the Epley formula: 1RM = w(1 + r/30), assuming repetitions > 1, where r represents the number of repetitions, and w is the weight of the load.

After the initial familiarization period, each exercise session from the third week onward consisted of six sets of barbell-back squats. Prior to the first set, participants performed a warm-up set of 12 repetitions, starting at 60% of their predicted 1RM, progressing to 10 repetitions at 70% of predicted 1RM in the second set, and finally 8 repetitions at 80% of 1RM in the last four sets. We encouraged participants to aim for one extra repetition in each of the last four sets, and the number of repetitions per set was based on the quality and intensity of their performance as well as their perceived exertion. The total training volume was calculated by multiplying the number of repetitions by the sets performed in 12 training sessions over 6 weeks. A rest period of 2 min was allowed between subsequent sets. For example, in the first formal intervention session (session 1), participant A used barbell weights of 10/12/14/14/14/14 kg, and the repetitions performed under each weight were 12/10/8 + 1/8 + 1/8 + 1/8 + 1. If she completed an extra repetition in each of the last four sets, her next training session would involve using weights of 12/10/10/10/10/10 kg and performing 12/10/10/10/10/10 repetitions. If she was able to achieve this for two consecutive sessions, the weights were increased by 2.5 kg for each set, resulting in weights of 12.5/14.5/16.5/16.5/16.5/16.5 kg and 12/10/8/8/8/8 repetitions per set. All participants’ progression loads were determined according to the progressive resistance training for healthy adults, with the content of the first to fourth sessions in six weeks shown in Table 2 as an example. All barbell back squat progression programs were shown in the supplementary S3.

**Table 2:** Barbell back squat progression.

Subjects	First session		Second session		Third session		Fourth session	
	Weight	R × S	Weight	R × S	Weight	R × S	Weight	R × S
1	30/32/34/34/34/34	12/10/9/9/8/8	30/32/34/34/34/34	12/10/9/9/9/9	30/32/34/34/34/34	12/10/10/10/10/10	30/32/34/34/34/34	12/10/10/10/10/10
2	40/44/46/46/46/46	12/10/9/9/8/8	40/44/46/46/46/46	12/10/9/9/9/9	40/44/46/46/46/46	12/10/10/10/10/10	40/44/46/46/46/46	12/10/10/10/10/10
3	40/44/46/46/46/46	12/10/9/8/8/8	40/44/46/46/46/46	12/10/9/9/9/9	40/44/46/46/46/46	12/10/10/10/10/10	40/44/46/46/46/46	12/10/10/10/10/10
4	46/50/54/54/54/54	12/10/9/8/8/8	46/50/54/54/54/54	12/10/9/9/9/9	46/50/54/54/54/54	12/10/10/10/10/10	46/50/54/54/54/54	12/10/10/10/10/10
5	40/44/46/46/46/46	12/10/9/9/8/8	40/44/46/46/46/46	12/10/9/9/9/9	40/44/46/46/46/46	12/10/10/10/10/10	40/44/46/46/46/46	12/10/10/10/10/10
6	40/44/46/46/46/46	12/10/9/9/8/8	40/44/46/46/46/46	12/10/9/9/9/9	40/44/46/46/46/46	12/10/10/10/10/10	40/44/46/46/46/46	12/10/10/10/10/10
7	50/56/60/60/60/60	12/10/9/8/8/8	50/56/60/60/60/60	12/10/9/9/9/9	50/56/60/60/60/60	12/10/10/10/10/10	50/56/60/60/60/60	12/10/10/10/10/10

Note: Repetitions × Sets = R × S, weight unite (kilogram); 1–7 represents each participant; The table shows the training volume (intensity, number of sets and times) completed by 7 participants in the barbell squat group in the first four training sessions.

### Measurements

The measurements were taken at baseline and 48 h after the final training session of the formal intervention. All individuals responsible for taking the measurements were unaware of the participants’ treatment status.

### Anthropometrics

Body height, weight, and body composition (including body fat percentage, lean body mass, minerals, and body water) were measured using InBody 770 (InBody Co, Ltd, Seoul, Korea) at baseline and 24 h after completing the last exercise session. Participants were instructed to wear light clothing and no shoes. They stood barefoot on the test platform with their feet in contact with the electrodes and their hands holding the test handle until the machine successfully measured their body composition. All personnel involved in the measurements were blinded to the participants’ treatment status.

### Strength measurement

Isokinetic knee extensor and flexor muscle peak torque of each leg were assessed using a dynamometer (PHYSIOMED CON-TREX-MJ, Schnaittach, Germany) both concentrically and eccentrically. Before the test, the subjects warmed up by riding a stationary bike (PRO2® SPORT TOTAL BODY, Tulsa, USA) at its easiest setting while seated. The seat and pedals were adjusted to ensure that the subjects were positioned properly to produce efficient power during the warm-up, which lasted 10 min. After a rest of three minutes, the participants were seated and secured to the dynamometer using torso and inactive thigh straps. Both legs were secured to the machine arm by two soft pads allowing comfortable knee movement. The testing protocol involved five successful trials for eccentric and concentric knee flexion and extension, where the participants produced a constant maximal effort. Concentric and eccentric peak torque was measured at an angular velocity of 60° per second between 0° and 90° of knee flexion. The trials were performed at 60°/s with self-determined maximum effort. Prior to the testing procedure, the participants were given instructions and two practice trials to familiarize themselves with the tasks. Verbal encouragement was given throughout each trial to ensure maximal effort.

### Measurement of muscle thickness

**Figure 2:**
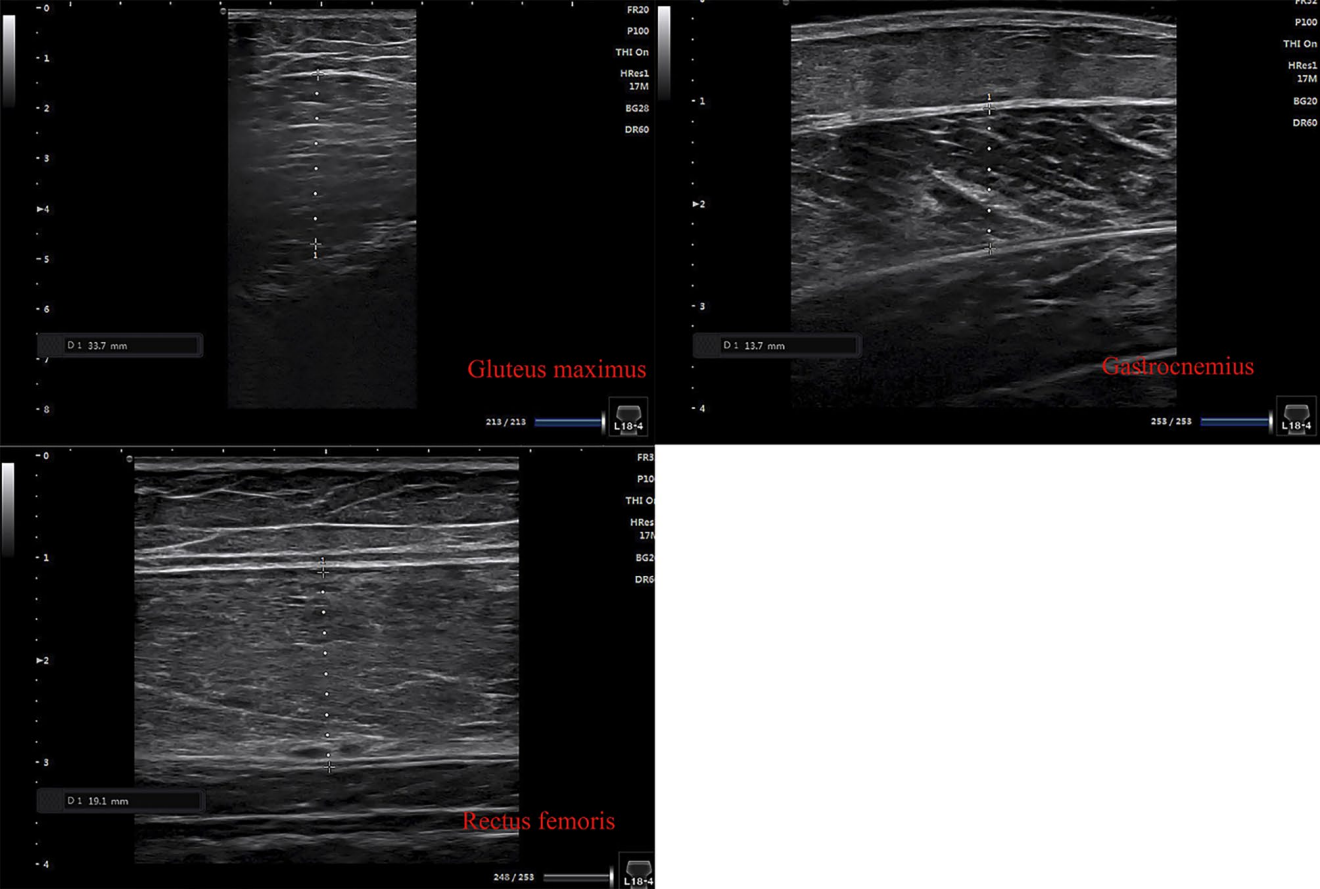
Muscle thickness images.

Muscle thickness was assessed using a B-mode ultrasound device (SONIMAGEHS1 musculoskeletal ultrasonic diagnostic system, Tokyo, Japan) at three anatomical sites: (a) gluteus maximus (the first third between the posterior superior iliac spine and the greater trochanter of the femur); (b) rectus femoris (a point two-thirds of the distance between the anterior–superior iliac spine and the superior tip of the patella on the anterior aspect of the thigh); and (c) gastrocnemius muscles (30% proximal between the lateral malleolus of the fibula and the lateral condyle of the tibia), as previously described^36–38^ (see Fig. 2). The scanning was performed with the subjects lying or sitting in a relaxed position with the dominant leg’s hip and knee. A 2.0–5.0 MHz scanning head was positioned perpendicularly on the skin surface. A water-based gel was applied, and minimal pressure was exerted on the probe to prevent muscle compression. The thickness of each muscle was measured as the maximum distance between the fascia layers on the B-mode image using the caliper function provided by the ultrasonography equipment. The same investigator conducted all ultrasound measurements. The images were stored on a data storage device for further analysis. All measurements were performed by the same operator, and the ICCs were 0.720 for gastrocnemius muscles, 0.587 for the rectus femoris, and 0.876 for the gluteus maximus.

### Covariates

To reduce the influence of other factors, we utilized a set of questionnaires to assess daily physical activity^35^, eating habits, movement quality, training volume, and perceived exertion of exercise during the intervention. The OMNI-Resistance Exercise Scale was used to rate perceived exertion for each exercise session, a widely used intensity measurement method in sports training along with heart rate^39^. To ensure training intensity consistency, perceived leg exertion was measured at the end of 3 and 6 sets, while perceived whole-body exertion was assessed at the end of 6 sets. The training volume (sets × repetitions) was calculated for all 12 training sessions (6 weeks). Participants were instructed to maintain their usual physical activity and eating habits, avoid additional resistance exercises, and abstain from muscle supplements like protein powder. If there were significant differences between the groups in the variables mentioned above or if they were significantly related to the measured outcomes, they were controlled for as covariates in the analysis.

### Statistical analysis

To test for normality assumptions, the Kolmogorov–Smirnov test was performed, and the Levene test was used to test for equal variance assumptions (p < 0.05). The independent-sample t-test was conducted if normality was assumed for baseline comparisons between groups in age, weight, body fat, maximum strength, and muscle thickness. The Mann–Whitney U test was conducted if normality was not assumed. For changes in all outcomes within the groups, a paired-sample t-test was used if normality and equal variance were assumed. If pre-test values were significantly different between groups at baseline, analysis of covariance (ANCOVA) was applied to test differences in post-test values between groups, including pretest values as a covariate, the posttest variable and the difference were taken as dependent variables respectively. Otherwise, the independent-sample t-test was conducted. The statistical analyses were performed using SPSS version 26 (SPSS, Inc., Chicago, IL, USA). The level of significance was set at p < 0.05, and all values are presented as mean ± standard deviation (SD).

### Ethics declarations

The studies involving human participants were reviewed and approved by Experiment of Sports and Health Promotion Research Center, College of P.E and Sports, Beijing Normal University of ethics committee. Written informed consent to participate in this study was provided by the all participants. Written informed consent was obtained from the individuals for the publication of any potentially identifiable images or data included in this article.

## Results

**Figure 3:**
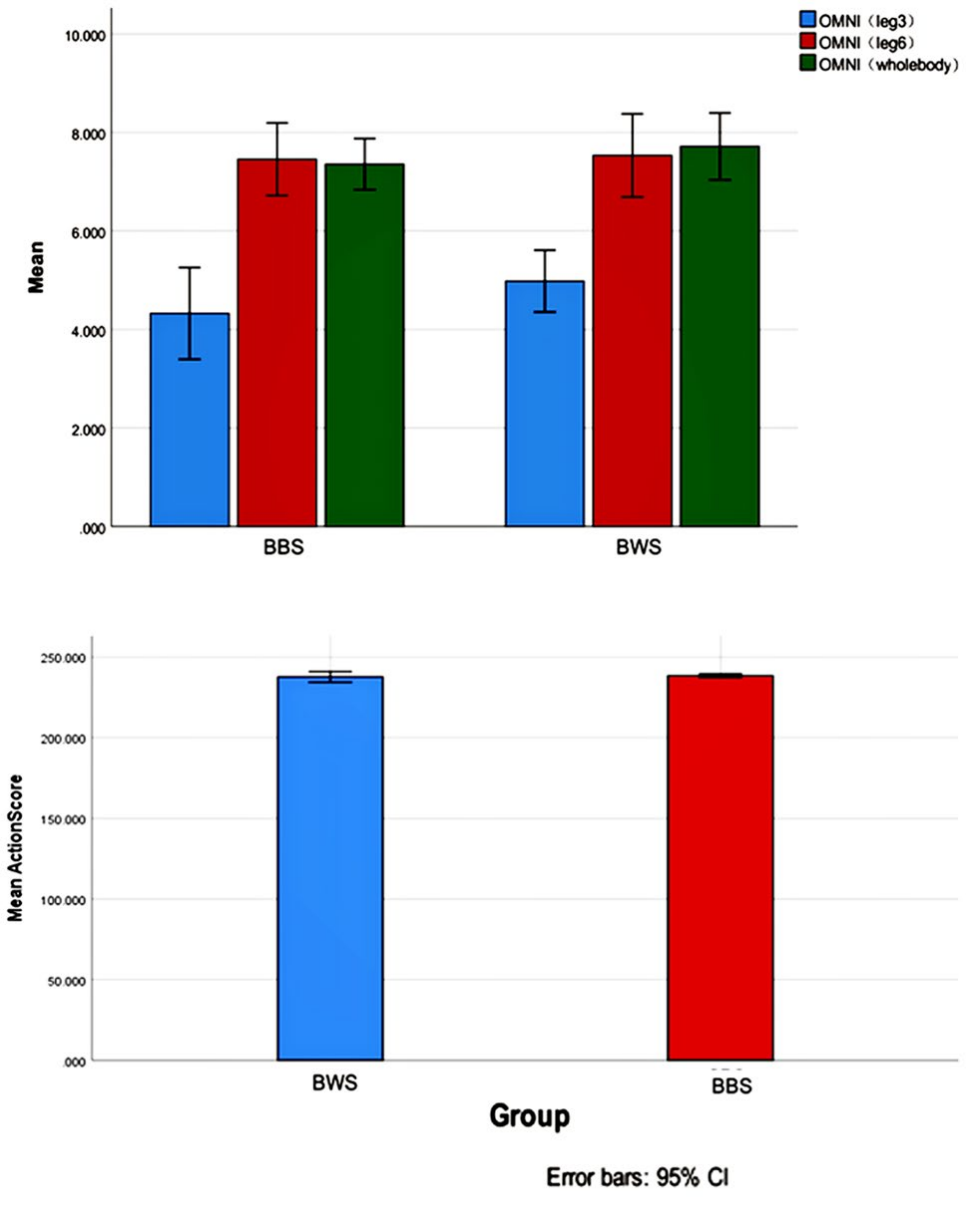
Perceive ratio and action score. Note. BBS, Barbell squat; BWS, Bodyweight squat; OMNI, OMNI-Resistance Exercise Scale (OMNI-RES) of perceived exertion; OMNI (leg 3), OMNI score of legs perceived exertion after completing 3 sets; OMNI (leg 6), OMNI score of legs perceived exertion after completing 6 sets; OMNI (leg 6), OMNI score of whole body perceived exertion after completing 6 sets.

Table 3 reports the baseline descriptive characteristics of the participants who completed the study. At baseline, the two groups were well matched in terms of age, height, weight, body fat, maximal strength, and muscle thickness (*p* > 0.05). After 6 weeks of training intervention, none of the subjects suffered injuries or experienced other adverse effects due to training. The total training volume (repetition × sets) between the two groups for 12 sessions showed no significant difference (F = 4.24, *p* = 0.142), although the bodyweight squat group (713.67 ± 7.09) was slightly higher than the barbell squat group (709.29 ± 1.89). There were also no significant differences between training intensity and motion quality during the intervention after 3 sets of perceived leg exertion (F = 2.52, *p* = 0.19), after 6 sets of perceived leg exertion (F = 0.16, *p* = 0.87), after 6 sets of perceived whole-body exertion (F = 0.09, *p* = 0.31), and activity quality (F = 2.38, *p* = 0.58) (Fig. 3).

**Table 3:** Baseline characteristics of participants (mean, standard deviation).

Group	Bodyweight group (n = 6)	Barbell-back group (n = 7)	F (*p*)
Age	19.25 ± 0.50	20.50 ± 1.00	0.23 (0.3)
Height (cm)	162.35 ± 4.47	167.48 ± 7.55	0.72 (0.15)
Weight (kg)	51.10 ± 4.39	58.66 ± 8.42	2.43 (0.07)
Body fat percentage (%)	24.18 ± 4.63%	28.66 ± 4.58%	0.16 (0.109)

Note: *independent-sample t-test, *p* ≤ 0.05. Descriptive statistics for all study participants (mean ± SD).

### Within-group differences in changes in maximum strength, muscle thickness, and body fat

After 6 weeks, the barbell-back squat group showed a significant decrease in body fat percentage (t (7) = 2.54, *p* < 0.05), while the bodyweight squat group did not (t (6) = 0.58, *p* = 0.59). Similar to the barbell-back squat group, the muscle thickness of the gastrocnemius (t (7) = − 3.37, *p* < 0.05) and gluteus maximus (t (7) = − 7.26, *p* < 0.001) increased significantly in the bodyweight group (t (6) = − 2.68, *p* < 0.05; t (6) = − 5.88, *p* < 0.05). However, neither group showed significant changes in the thickness of the rectus femoris (Barbell-back: t (7) = − 1.79, *p* = 0.12; Bodyweight: t (6) = − 2.59, *p* = 0.05) (Table 4).

**Table 4:** Effects on lower limbs muscle thickness.

Muscle thickness (mm)	Bodyweight group			Barbell-back group			Between
	Pre	Post	*p*	Pre	Post	*p*	*p*
Gastrocnemius	14.20 ± 1.49	16.20 ± 2.14	0.04	16.13 ± 2.93	18.73 ± 2.40	0.02	0.82
Rectus femoris	14.73 ± 2.14	17.00 ± 1.52	0.05	17.36 ± 3.14	19.46 ± 3.27	0.12	0.54
Gluteus maximus	28.15 ± 3.72	33.50 ± 2.34	0.00	29.31 ± 4.67	33.44 ± 4.50	0.00	0.91

Note: Pre- and Post-*Paired Sample T-test, *p* ≤ 0.05; Between Group-*independent-sample t-test, *p* ≤ 0.05. Descriptive statistics for all study participants (mean ± SD).

Note: 1 was pre, 2 was post.

**Figure 4:**
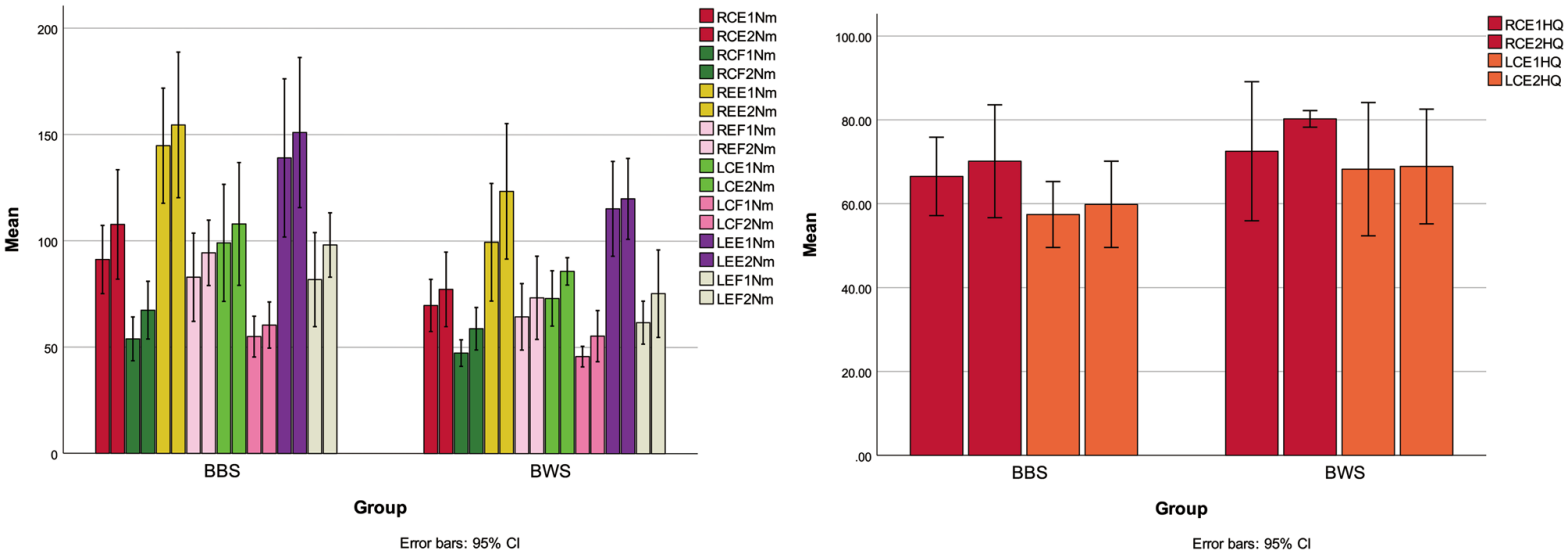
Pre and post on isokinetic peak torque. Note. RCE, Right Knee Concentric Peak Torque of Extensor; RCF, Right Knee Concentric Peak Torque of Flexor; REE, Right Knee Eccentric Peak Torque of Extensor; REF, Right Knee Eccentric Peak Torque of Flexor; LCE, Left Knee Concentric Peak Torque of Extensor; LCF, Left Knee Concentric Peak Torque of Flexor; LEE, Left Knee Eccentric Peak Torque of Extensor; LEF, Left Knee Eccentric Peak Torque of Flexor; Nm, Newton meter; RCHQ, The Mean Concentric Peak torque of Hamstring-to-Quadriceps Ratio in Right Knee; LCHQ, The Mean Concentric Peak Torque of Hamstring-to-Quadriceps Ratio in Left Knee; 1 was pre, 2 was post.

In the barbell back squat group, Fig. 4 depicted the changes in muscle strength. For the right knee, the concentric peak torque of flexors (RCF) (t (7) = − 4.30, *p* < 0.05) and extensor (RCE) (t (7) = − 2.60, *p* < 0.05), as well as the eccentric peak torque of flexor (REF) (t (7) = -2.51, *p* < 0.05) and extensor (REE) (t (7) = − 0.89, *p* = 0.41), were significantly increased. For the left knee, the concentric peak torque of the flexor (LCF) (t (7) = − 3.65, *p* < 0.05) and extensor (LCE) (t (7) = − 3.27, *p* < 0.05), as well as the eccentric peak torque of the flexor (LEF) (t (7) = − 2.76, *p* < 0.05), were significantly changed, but not the eccentric peak torque of the extensor (LEE) (t (7) = − 1.59, *p* = 0.16). The mean concentric peak torque of the right knee H/Q-ratio (RCHQ) was t (7) = − 1.22,* p* = 0.27, while the mean concentric peak torque of the left knee H/Q-ratio (LCHQ) was t (7) = − 0.67,* p* = 0.53 (Table 5).

**Table 5:** Effects of maximal strength, H/Q ratio and body fat in two types of progressive resistance training.

Group	Bodyweight squat			Barbell-back squat			Between difference
Items	Pre	Post	*p*	Pre	Post	*p*	*p*
Isokinetic (Nm)	60°/s	60°/s		60°/s	60°/s		
RCE	69.67 ± 11.70	77.23 ± 16.71	0.26	91.17 ± 17.34	107.67 ± 27.82	0.04	0.34
RCF	47.27 ± 5.97	58.73 ± 9.51	0.01	53.84 ± 11.16	67.34 ± 14.70	0.00	0.51
REE	99.40 ± 26.36	123.37 ± 30.40	0.01	144.70 ± 29.28	154.49 ± 37.04	0.41	0.83
REF	64.33 ± 14.90	73.27 ± 18.65	0.10	82.87 ± 22.43	94.36 ± 16.63	0.05	0.17
LCE	72.98 ± 12.39	85.73 ± 6.12	0.04	99.03 ± 29.77	107.93 ± 31.27	0.01	0.89
LCF	45.67 ± 4.61	55.32 ± 11.46	0.06	54.97 ± 0.35	60.37 ± 0.18	0.01	0.67
LEE	115.15 ± 21.26	119.87 ± 18.13	0.34	139.01 ± 40.20	150.96 ± 38.17	0.16	0.77
LEF	61.65 ± 9.63	75.28 ± 19.59	0.06	81.76 ± 23.95	98.09 ± 16.35	0.03	0.18
H/Q ratio (%)	60°/s	60°/s		60°/s	60°/s		
RCHQ	72.52 ± 15.80%	80.23 ± 1.88%	0.32	66.51 ± 10.12%	70.14 ± 14.55%	0.27	0.25
LCHQ	68.25 ± 15.13%	68.88 ± 13.03%	0.93	57.43 ± 8.50%	59.86 ± 11.12%	0.53	0.51
Body fat (%)	24.18 ± 4.63%	24.02 ± 4.78%	0.68	28.66 ± 4.58%	24.96 ± 5.91%	0.04	0.10

Note: Pre- and Post-*Paired Sample T-test, *p* ≤ 0.05; Between Group-*independent-sample t-test, *p* ≤ 0.05. Descriptive statistics for all study participants (mean ± SD). Right Knee Concentric Peak Torque of Extensor, RCE; Right Knee Concentric Peak Torque of Flexor, RCF, Right Knee Eccentric Peak Torque of Extensor, REE; Right Knee Eccentric Peak Torque of Flexor, REF; Left Knee Concentric Peak Torque of Extensor, LCE; Left Knee Concentric Peak Torque of Flexor, LCF; Left Knee Eccentric Peak Torque of Extensor, LEE; Left Knee Eccentric Peak Torque of Flexor, LEF. The Mean Concentric Peak torque of Hamstring-to-Quadriceps Ratio in Right Knee, RCHQ; The Mean Concentric Peak Torque of Hamstring-to-Quadriceps Ratio in Left Knee, LCHQ.

In the group performing bodyweight squats, there were changes observed in the isokinetic torque of the right knee. Specifically, the concentric peak torque of the flexor (RCF) showed a statistically significant difference of t (6) = − 3.73 (*p* < 0.05), while the concentric peak torque of the extensor (RCE) did not show a significant difference at t (6) = − 1.26 (*p* = 0.26). The eccentric peak torque of the flexor (REF) showed a trend towards significance at t (6) = − 2.03 (*p* = 0.10), and the eccentric peak torque of the extensor (REE) showed a significant difference at t (6) = − 4.41 (*p* < 0.05). As for the left knee, the concentric peak torque of the flexor (LCF) showed a trend towards significance at t (6) = − 2.43 (*p* = 0.06), while the concentric peak torque of the extensor (LCE) showed a significant difference at t (6) = − 2.85 (*p* < 0.05). The eccentric peak torque of the flexor (LEF) also showed a trend towards significance at t (6) = − 2.44 (*p* = 0.06), while the eccentric peak torque of the extensor (LEE) did not show a significant difference at t (6) = − 1.06 (*p* = 0.34). Regarding the H/Q ratio, the mean concentric peak torque of the knee H/Q ratio (RCHQ) did not show a significant difference at t (6) = − 1.11 (*p* = 0.316), and the mean concentric peak torque of the knee H/Q-ratio (LCHQ) also did not show a significant difference at t (6) = − 0.96 (*p* = 0.93).

### Between-group differences in changes in maximal strength, muscle thickness, and body fat

**Figure 5:**
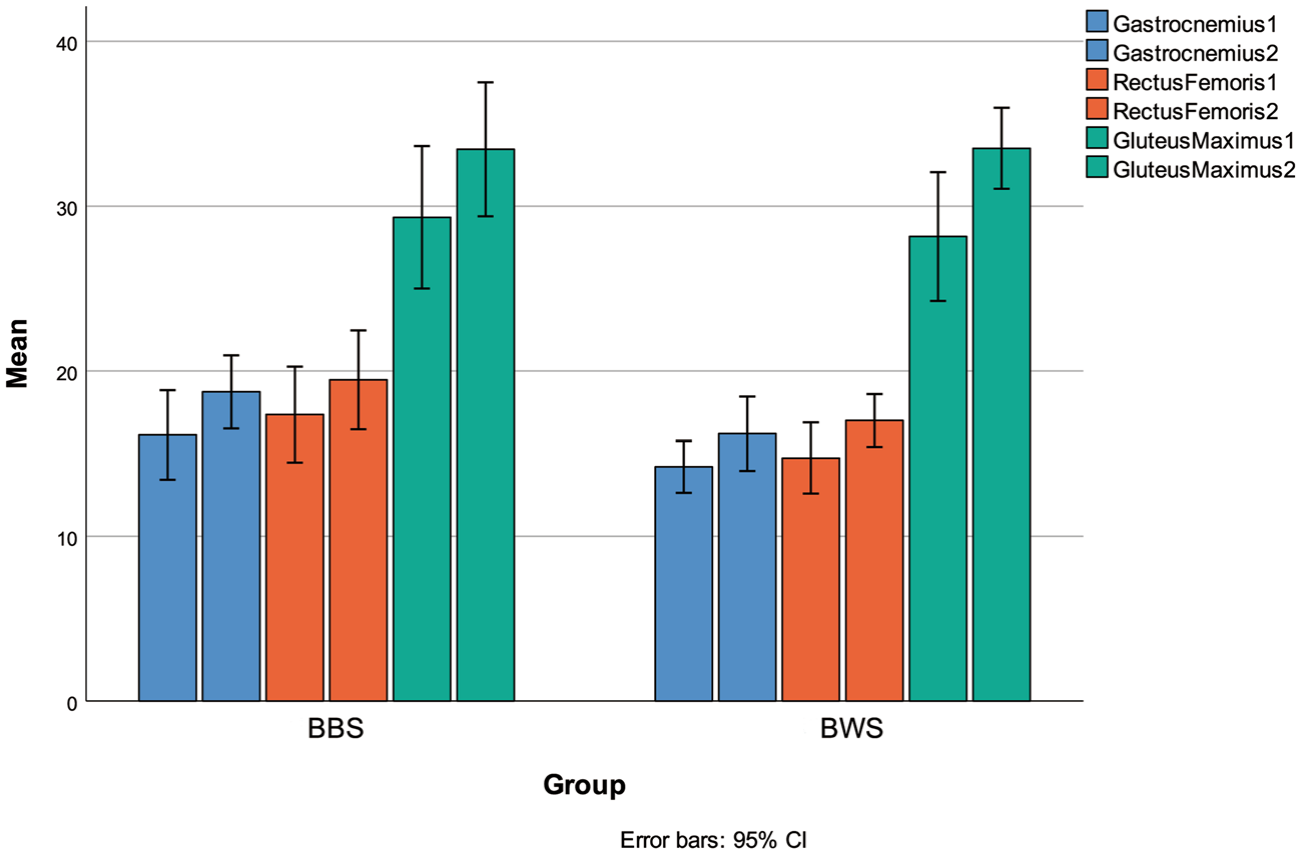
Pre and post of two groups on muscle thickness. Note: 1 was pre, 2 was post.

After 6 weeks, there were no significant differences in peak torque of knee extensor and flexor, as well as the H/Q ratio between the two groups (measured in N·m) (*p* ≥ 0.05, see Table 6). No significant differences were observed for lower limb muscle thickness between the two groups (see Fig. 5). The gastrocnemius thickness of both groups was F = 0.05 (*p* = 0.84), the rectus femoris thickness was F = 1.54 (*p* = 0.28), and the gluteus maximus thickness was F = 1.61 (*p* = 0.27). As shown in Table 4, there were no significant differences between groups for body weight (F = 0.84, *p* = 0.41) and body fat (F = 0.44, *p* = 0.55). Additionally, there were no significant differences in the change value of isokinetic peak torque, H/Q ratio, and body fat between pre-and post-measurements (see Table 6).

**Table 6:** The D-value of isokinetic peak torque, H/Q ratio and body fat in two types of progressive resistance training.

Group	Bodyweight group (n = 6)	Barbell-back group (n = 7)	*p*
Body fat percentage (%)	0.27 ± 0.90%	3.79 ± 3.66	0.10
Rectus femoris (mm)	2.00 ± 1.83	2.74 ± 2.35	0.93
Gluteus maximus (mm)	2.27 ± 2.14	2.10 ± 3.11	0.44
Rectus femoris (mm)	5.35 ± 2.23	4.13 ± 1.51	0.26
Isokinetic (Nm)			
RCE	7.57 ± 14.72	16.93 ± 17.21	0.69
RCF	11.47 ± 7.54	10.64 ± 6.99	0.63
REE	23.97 ± 13.3	9.93 ± 29.15	0.07
REF	8.98 ± 10.84	11.49 ± 12.12	0.95
LCE	12.75 ± 10.96	8.90 ± 7.21	0.34
LCF	9.65 ± 9.71	5.40 ± 3.91	0.29
LEE	4.72 ± 10.94	11.94 ± 19.91	0.37
LEF	14.17 ± 13.04	16.33 ± 15.68	0.47
H/Q ratio (%)			
RCHQ	3.18 ± 17.56%	6.06 ± 5.56%	0.18
LCHQ	1.80 ± 9.39%	1.31 ± 15.72%	0.50

Note: D-value = post-test value-pre-test value; Pre and Post-*Paired Sample T-test, *p* ≤ 0.05; Between Group-*independent-sample t-test, *p* ≤ 0.05. Descriptive statistics for all study participants (mean ± SD).

## Discussion

The aim of this study was to compare the impact of two different progressive squat programs (bodyweight and barbell-back squat) on body fat, lower limb muscle thickness, and strength in sedentary women over a period of 6 weeks. It examined the specific effects of different postures and angles of bodyweight squats on lower limb muscle strength compared to barbell-back squats. Despite the fact that the number of training repetitions for both groups remained essentially the same, the training mode that increases the level of movement difficulty as the load intensity was found to be just as effective as gradually increasing the barbell weight as the load intensity, which can help increase knee muscle strength and muscle hypertrophy levels for the subject. The study’s main findings revealed that both groups exhibited similar increases in lower limb isokinetic peak torque and muscle thickness, but changes in body fat differed between the two groups.

Previous studies have shown that bodyweight squat mode, which focuses on increasing the number of repetitions, can improve the total training volume by increasing the number of repetitions under constant load intensity (bodyweight)^22^. However, this progressive strategy may be less effective in improving maximum strength and more effective for improving muscle endurance. Existing evidence indicates that training at 80% 1RM or above intensity is more effective for increasing maximum strength than training at high repetitions (training to failure)^40,41^. Existing studies have also shown that varying squat posture and joint angles can lead to significant load pressure differences in different muscle groups. For example, research has shown that knee flexion of 0°–50° has relatively minimal pressure around the knee, while increasing knee flexion angle activates the quadriceps, posterior femoris, and gastrocnemius muscles to a greater extent^38^. The selection of different squatting positions can significantly affect squat angles and the distribution of resistance load. Single-leg squats and double-leg squats have been shown to have significant differences in the maximum flexion angle of the knee and hip joints during squats^39^. The split squat, on the other hand, exhibits a load pressure distribution difference of more than 25% between the legs^40^. Even in barbell squats, there is a significant difference in trunk pressure when the knee exceeds the tip of the foot compared to when it does not, even when squatting to the same depth^41^. Previous studies have shown that Bulgarian squats stimulate antagonistic and core muscles significantly more than double-leg squats^42^. This study further demonstrates that bodyweight squat training can increase training intensity by incorporating different body and leg postures with varying difficulties and angles, allowing for more effective improvements in muscle strength and hypertrophy.

With regard to muscle strength, both groups of untrained women experienced comprehensive improvements, including in right knee concentric peak torque of flexor, right knee eccentric peak torque of extensor, and left knee concentric peak torque of extensor. This study further proved that bodyweight squats, using body-weight as the load intensity and focusing on multiple unilateral squats combinations, can achieve a similar increase in knee joint strength as double-leg barbell squats with 60–80% 1RM in short term. Results about the bodyweight squat in this study were also consistent with those of previous studies, which showed that various leg squats can significantly stimulate the quadriceps and hamstrings^42,43^. The H/Q ratio, an important index for knee stability and rehabilitation, was maintained at a high level before and after training in the bodyweight squat group, which is comparable to a typical H/Q ratio of a healthy knee. This may be because bodyweight squats mainly focused on unilateral squats (F–J) in the middle and late stages of training, such as Bulgarian squats, Skating squat, and single-leg squat.

Meanwhile, previous evidence have shown significant differences in H/Q ratios between dominant and non-dominant legs in young women^44^. However, after bodyweight squat intervention in the present study, the symmetry of lower limb H/Q ratio of the subjects did not significantly change or increase the difference. The reason may be that more unilateral squat exercises can help reduce the dependence of training stimulation on the dominant leg, leading to a better balance of muscle strength in both legs. These results demonstrated that bodyweight squat is a suitable approach to maintaining knee stability. In addition, even in a comparison of unilateral and bilateral squats using barbell, unilateral squats using a lower load intensity (28 kg) were found to produce similar stimulation to the thigh, calf, hip, and abdominal muscles, but with less stress on the spine, compared with double-leg squats (135 kg) using a higher load intensity^45^. Therefore, for sedentary women, bodyweight squats that focus on unilateral squats can effectively improve and balance the strength of the muscles around the knee joint while avoiding greater spinal pressure, compared with traditional double-leg squats with barbells.

In terms of muscle hypertrophy, early studies generally agree that neural adaptation plays a dominant role during the first 6–7 weeks of training, while muscle hypertrophy changes very little during this period^47^. However, a large body of research has shown that significant muscle hypertrophy responses can occur in the early stage (6–12 weeks) of training with appropriate frequency, intensity, and volume^48,49^. Moreover, the number of repetitions has been found to be significantly correlated with muscle hypertrophy. Previous studies have demonstrated hypertrophy occurrence after 6 weeks of squat training with 3–8 repetition maximums in untrained women or men^50,51^. In this study, a 6-week progressive squat training program, which increased the load while completing the maximum number of repetitions, significantly improved the lower limb muscle circumference in both groups. Previous research has also found significant effects on knee joint isokinetic muscle strength and thigh muscle circumference in college students after a 6-week program of front lunges and squats, as well as two bodyweight squats with blood flow restriction, without reaching maximum repetitions^49^. These findings suggest that short-term bodyweight squat training with 8–12 maximum repetitions under different variations (unilateral and bilateral) can have significant positive effects on promoting muscle hypertrophy, consistent with previous studies.

Muscle cross-sectional areas had a significant relationship with strength-velocity characteristics of the whole muscle and greater force production^46,47^. The muscle cross-sectional area of both groups in the present study showed similar significant improvements without significant between-group difference. This finding was consistent with a previous study showing that muscle cross-sectional area has a positive relationship with maximal forces^46^. Moreover, in the current study, the gluteus maximus exhibited a greater increase in cross-sectional area than the rectus femoris and gastrocnemius in the bodyweight squat group. This was likely due to the prevalence of lunge and single-leg squat exercises in this group^27^, which aligns with previous research indicating higher EMG activity in the gluteus maximus during these exercises compared to the rectus femoris and gastrocnemius. In the barbell-back squat group, there was significant improvement in muscle cross-sectional area for both the gluteus maximus and gastrocnemius, while the rectus femoris did not exhibit a significant improvement. This could be attributed to the focus on controlling the knee joint in barbell squat training to transfer more force to the hip, as limiting forward displacement of the knee joint can enhance this effect^18^. Additionally, gluteus maximus activation increases proportionally with load in barbell squatting^47^, potentially explaining the greater improvement in gluteus maximus cross-sectional area in this group compared to the rectus femoris.

Finally, participants in the barbell-back squat group experienced a significant reduction in body fat percentage without a change in body weight, consistent with previous findings that resistance training can lead to rapid decreases in body fat in untrained women^48^. The higher starting body fat percentage in the barbell group may have contributed to the significant differences in improvement seen between groups.

## Conclusion

Considering the importance of strength, particularly lower limb strength, for sedentary women, this randomized controlled trial provides evidence supporting the feasibility and effectiveness of progressive bodyweight squat training for improving knee joint strength and muscle circumference growth in sedentary young women over a 6-week period. These findings extend existing research on lower extremity strength promotion in the general population through strength training methods. Based on the resistance training methods that incorporate different unilateral squat positions using bodyweight, it may have short-term effects on the development of strength and the muscular system, from knee strength to lower extremity muscle circumference. Therefore, we suggest that bodyweight squat training can be used as an alternative to traditional resistance training for sedentary young women.

### Practical implications

The progressive bodyweight squat program that was developed can be widely applied to the general population. It is also valuable for therapists and practitioners who have back problems, as it can improve lower limb muscle strength without placing additional burden on the back. This is particularly useful because back problems are often accompanied by reduced strength in the lower limbs.

### Limitations and future research

Our study has some limitations that require attention. Firstly, while participants were instructed to maintain their regular diet, the lack of control over their diet could potentially influence the accretion of muscle mass and serve as a confounding factor. Secondly, it would be worthwhile to investigate the long-term effects of the two training sessions on body fat, muscle strength, and thickness by conducting studies with longer training periods. Thirdly, the small number of participants in our study limits the generalizability of the results, and further research is needed to investigate the training effects on a larger sample size. Additionally, it would be beneficial to examine the impact of bodyweight squat on the stability of core and lower limb joints, which could be a valuable area for future research.

### Supplementary Information

Supplementary Information 1.

Supplementary Information 2.

Supplementary Tables.

## Data Availability

The datasets analyzed during the current study are available from the corresponding author on reasonable request.
