# Supplementary material for: Effects of progressive body-weight versus barbell back squat training on strength, hypertrophy and body fat among sedentary young women
Source: Sci Rep. 2023 Aug 19;13:13505. doi: 10.1038/s41598-023-40319-x (PMC10439966; doi:10.1038/s41598-023-40319-x)

Movements scoring guide

Notes:

1、The illustration is divided into 3 parts, namely the warm-up part, the intervention part and the stretch part. The intervention part is divided into self-weight squats and barbell squats.

2、 The required score of each exercise action is the same as that of the bottom (if there is 1 × 2, note that the score value of each action is not 1).

一、 Warm-up part:

1. head stretch

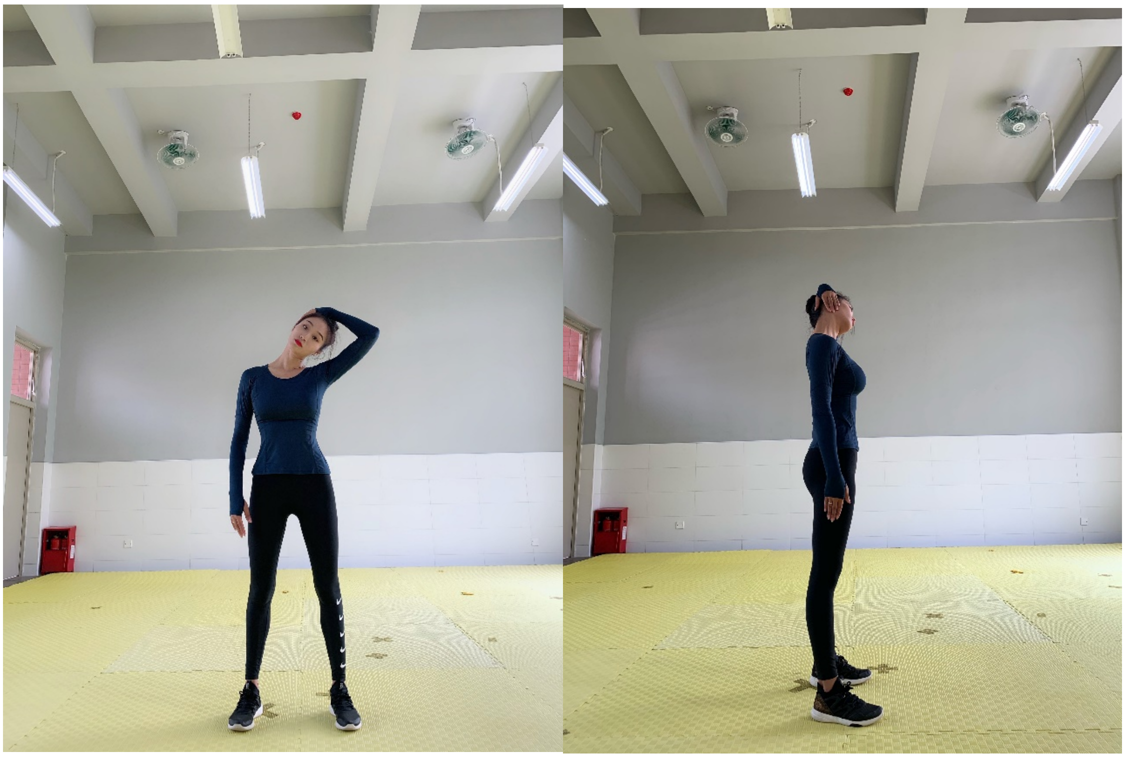

Left side (base point 0.24) :

- Shoulders relaxed;
- Place your right hand over the left ear of your head;
- Press your head down with your hand against your right shoulder;
- The head and the right hand form a counterforce.

Right side (base 0.24) :

- Shoulders relaxed;
- Keep your left hand over the right ear of your head;
- Press your head down against your left shoulder;
- The head and the left hand form opposing forces.

Rear stretch **(0.6) :**

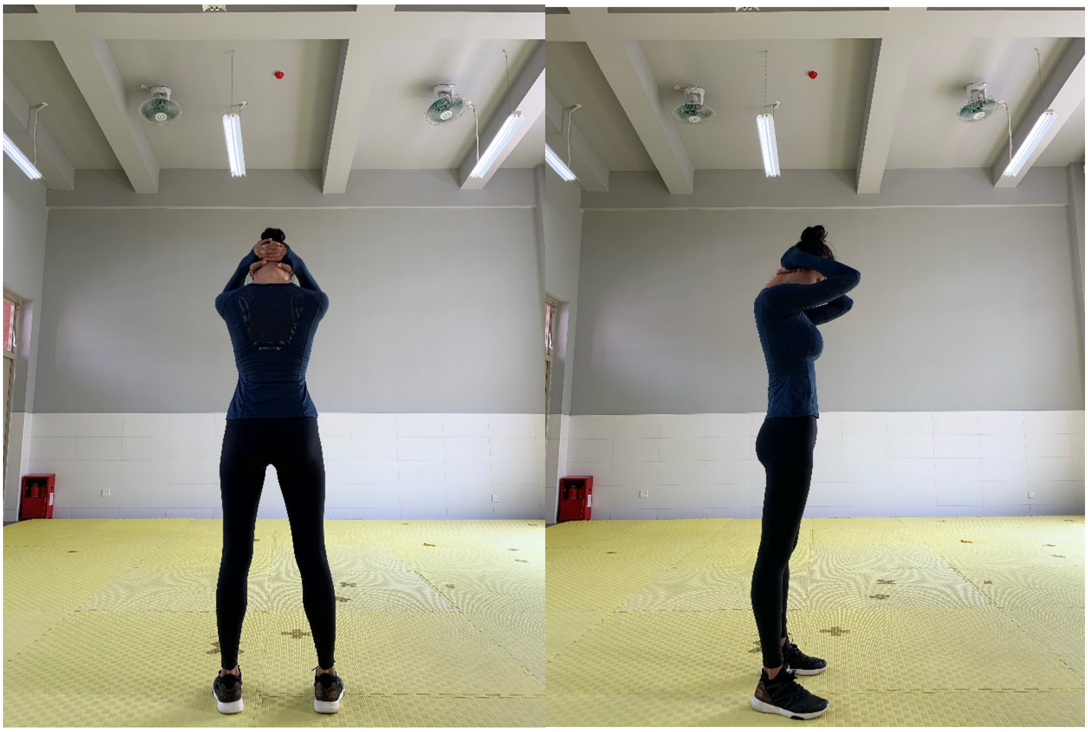

- Chin close to collarbone.

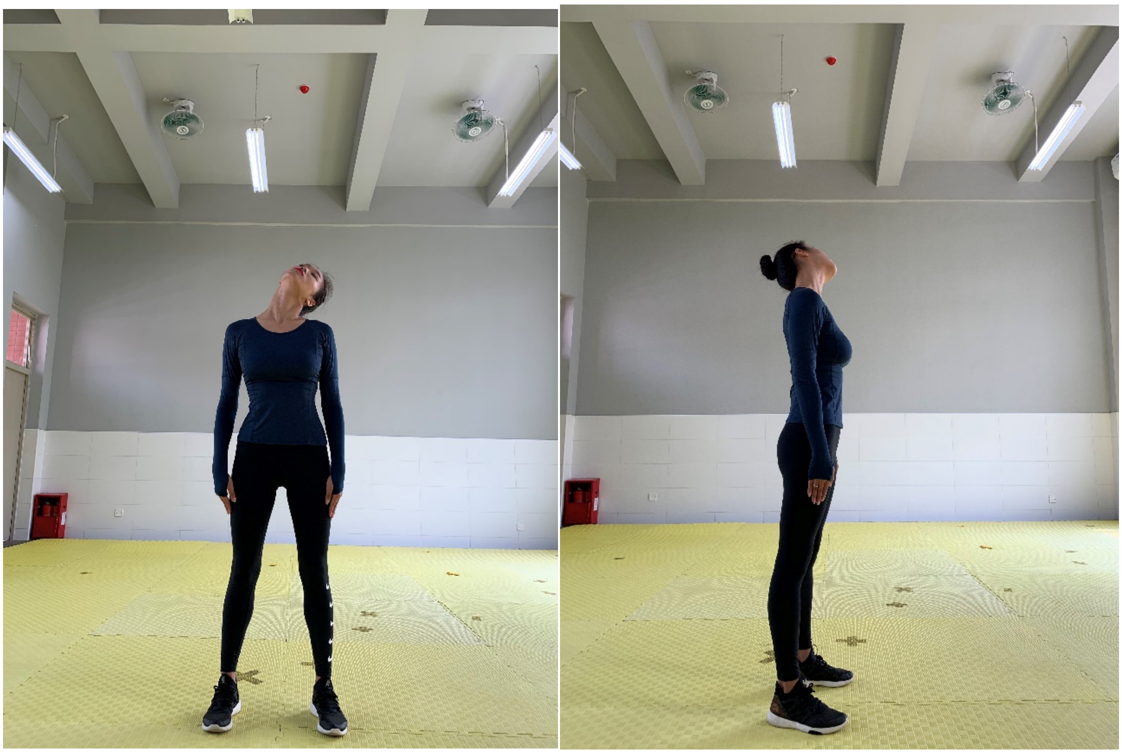

Left front (base score 0.4) :

- Chin up;
- And tilt your cheeks to the right.

Right front side (base point 0.4) :

- Chin up;
- And tilt your cheeks to the left.

2. Shoulder girdle (base point 0.06)

- Side lift with both arms flat;
- Stand your palms straight with your fingers together;
- When winding the ring, take the shoulder as the axis, take the position of the palm when the side lift as the center of the circle, drive the arm up and down 15 degrees to draw a circle;
- The muscles around the shoulder exert uniform force, holding each circle for about 1 second.

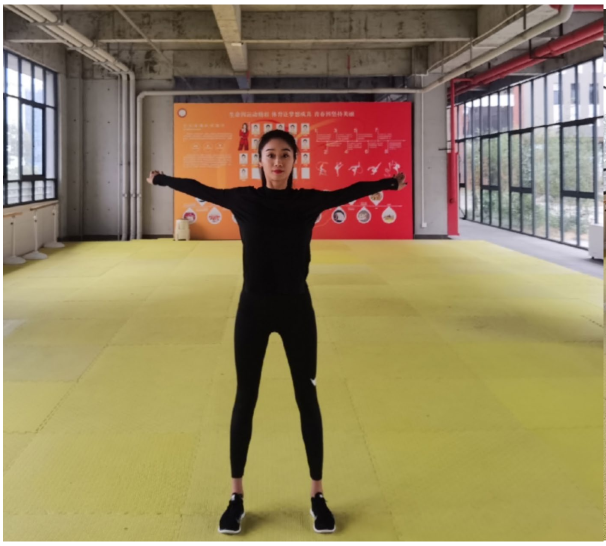
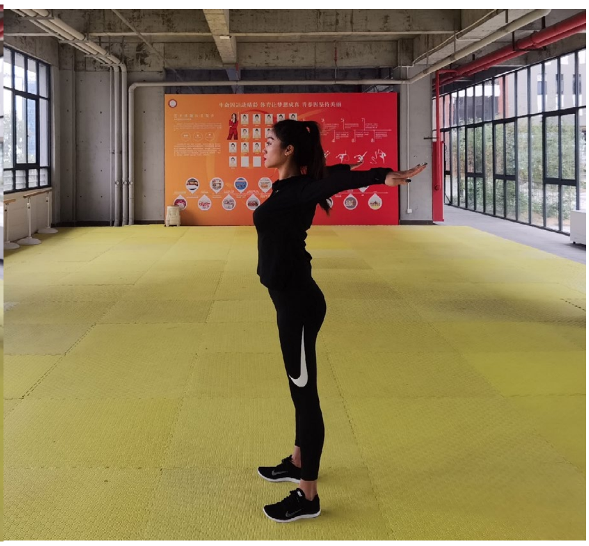

3. Crawl worm stretch (bottom minute 0.12)

- Stand with your feet shoulder-width apart, bend over with straight legs, support the floor with your hands at the nearest point of your feet, and keep your hands shoulder-width apart;
- Move your arms forward until your body is parallel to the floor;
- Keeping your legs straight, bring your palms back in turn to the point closest to your feet and stand upright;
- The main muscles in black are latissimus dorsi, pectoralis major, anterior deltoid, brachialis, etc.

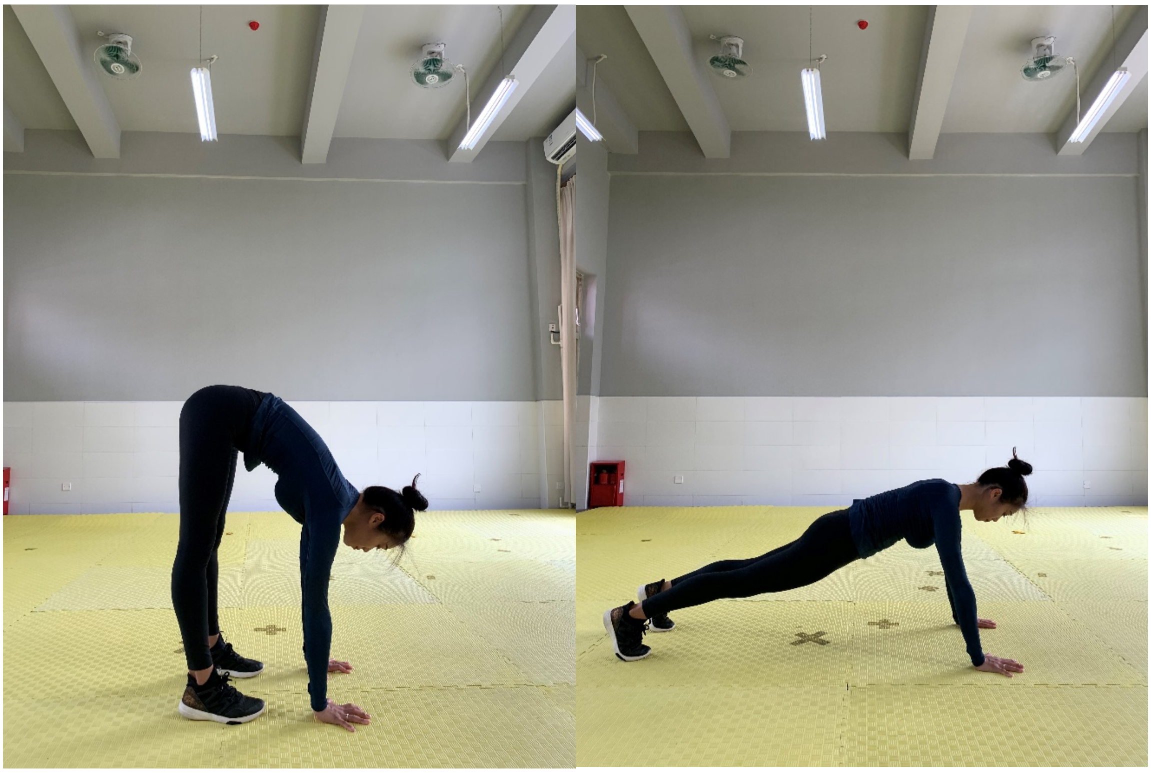

4. Jumping Jack (base point 0.0375)

- Feet together, chest out, stomach in, waist and back straight, in a standing position;
- High five with your arms straight up;
- Jump slightly and open your legs 60 degrees;
- Extend your arms straight down to your sides;
- At the same time, keep your legs together and land with your knees slightly bent and cushioned;
- When the arms swing, the shoulder, upper back and chest muscles exert force;
- When jumping, push off the ground through your thighs, calves, and front balls of your feet.

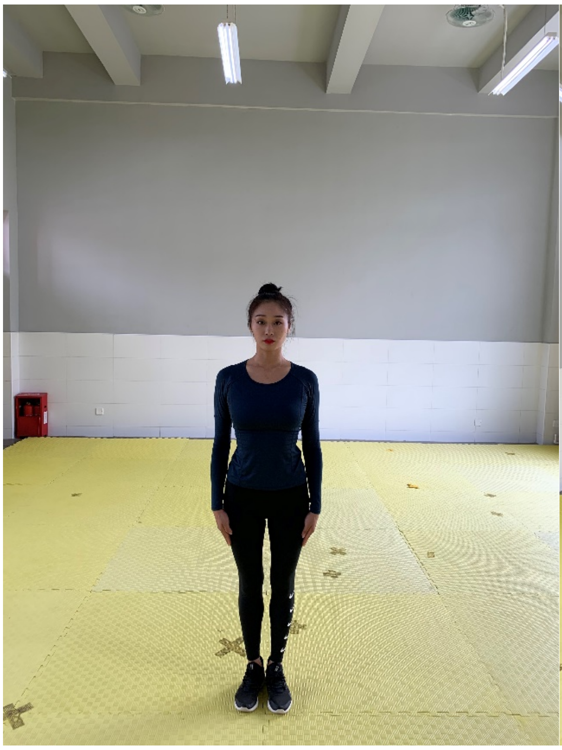
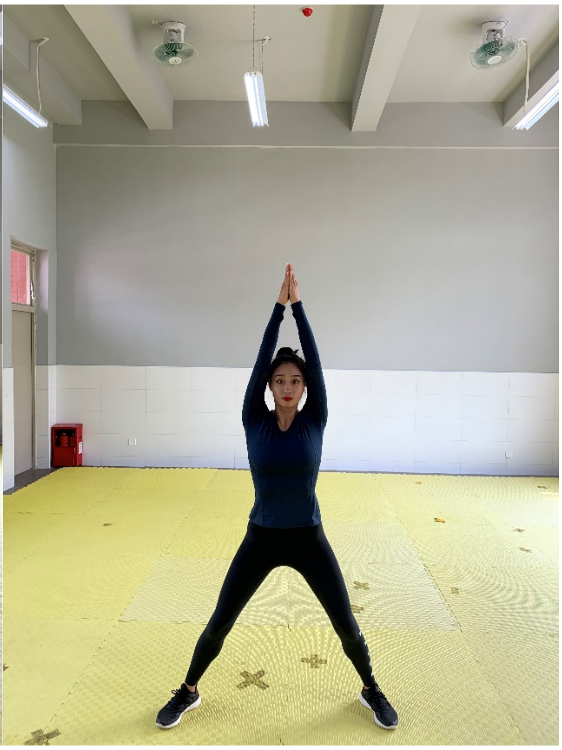

5. balance stand (bottom part 0.0375 × 2)

Left leg stand:

- Keep your feet together, chest out, stomach in, waist and back straight, arms raised sideways, in a standing position;
- Keep your left leg upright at all times;
- Lean forward with your upper body on the hip axis;
- Raise the right leg until it is in line with the body and parallel to the floor and hold for 1 second;
- Keep your arms horizontally raised with your shoulders, upper back and chest;
- The muscles around the back and spine exert force to control the body falling and lifting;
- The gluteus maximus and leg muscles work to support and lift the legs.

To stand on your right leg:

- Ditto

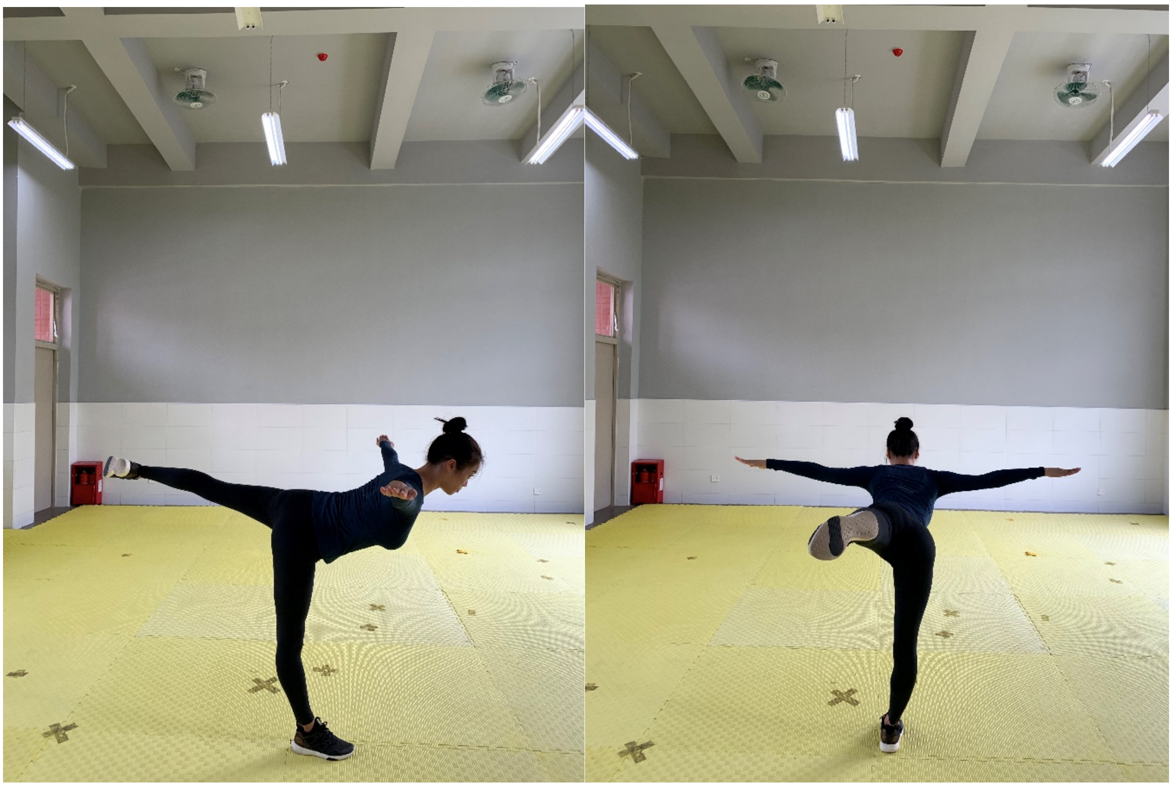

6. Kneeling position push-ups (0.1 base point)

- Lie prone on the ground with your calves crossed and your knees on the ground with your hands slightly wider than shoulder width and your straight arms at your sides;
- Bend your elbows down until your upper arms are parallel to the floor;
- The Angle between the coronal arm and torso should be 60 degrees;
- Shoulder, waist and thigh are in a straight line when they are at their lowest point;
- The anterior deltoid tract, triceps, chest muscles, and waist and abdomen exert force.

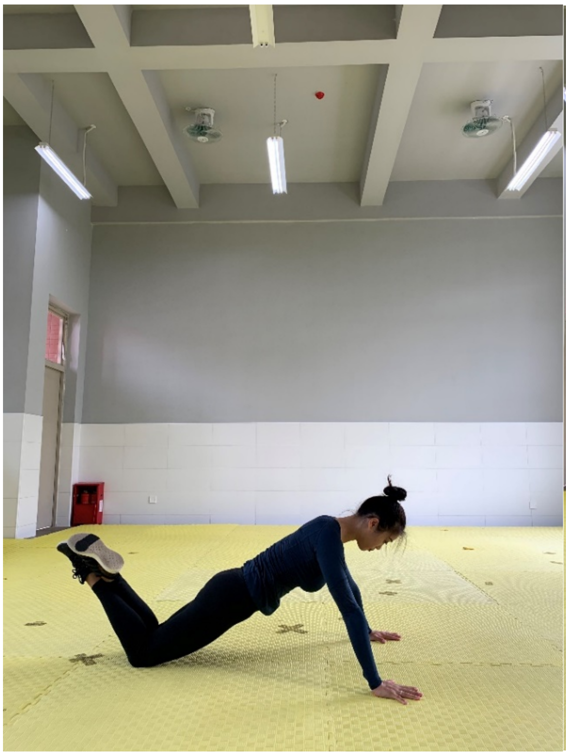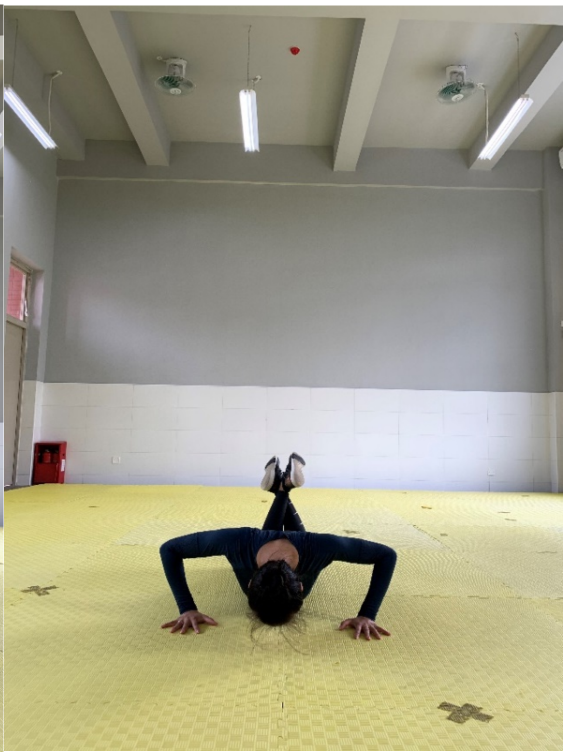

7. Prone twist (base point 0.1)

Left arm up:

- Hands and feet shoulder-width apart, in a straight-arm push-up position, shoulders, waist, and thighs always in a straight line;
- Support the right arm, raise the left arm, arms in a straight line, and perpendicular to the ground;
- Raise your left arm until it is in a straight line and perpendicular to the floor and return to a push-up position.
- When lifting the right arm arm and shoulder support force to keep the upper limb stable;
- Push the shoulders, upper back and chest to lift the arms.

Right arm lift:

- Ditto

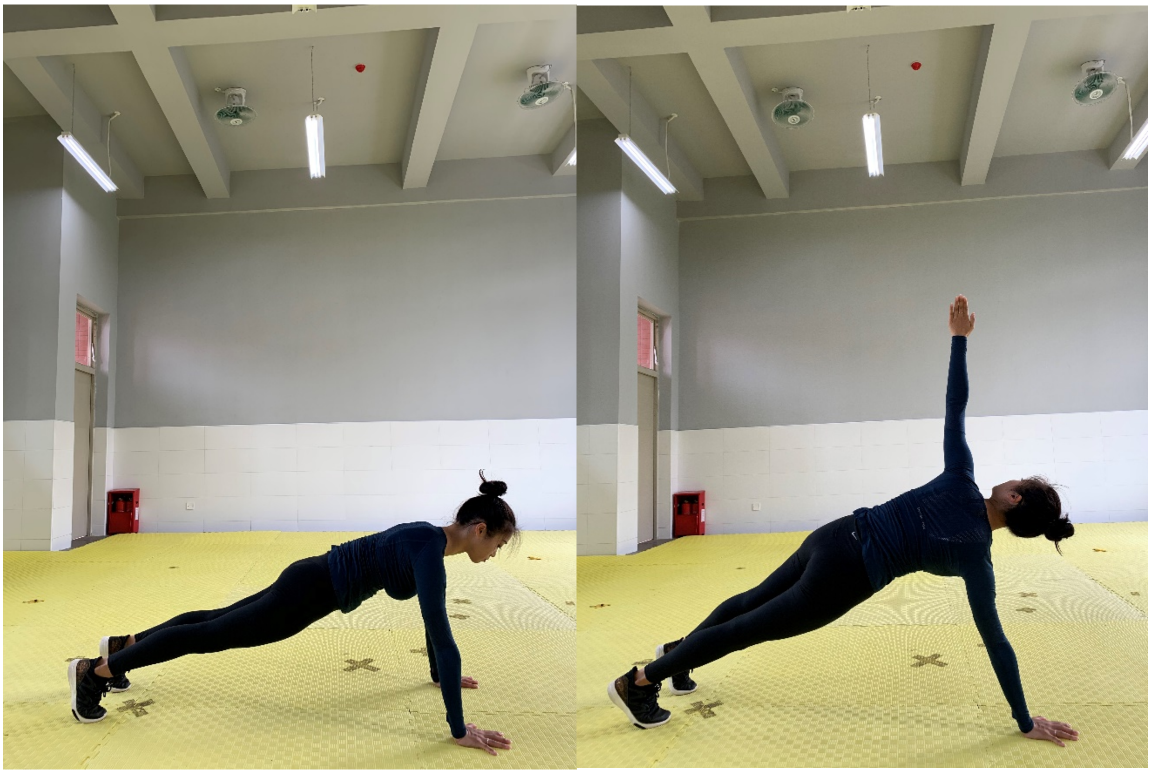

8. Plank (bottom score 1)

- Hands and feet shoulder-width apart in a bent elbow push-up position;
- Shoulder, waist and ankle joints in the same straight line;
- The big arms are perpendicular to the ground;
- Exert force on the shoulders and waist and abdomen;
- Lower limb force.

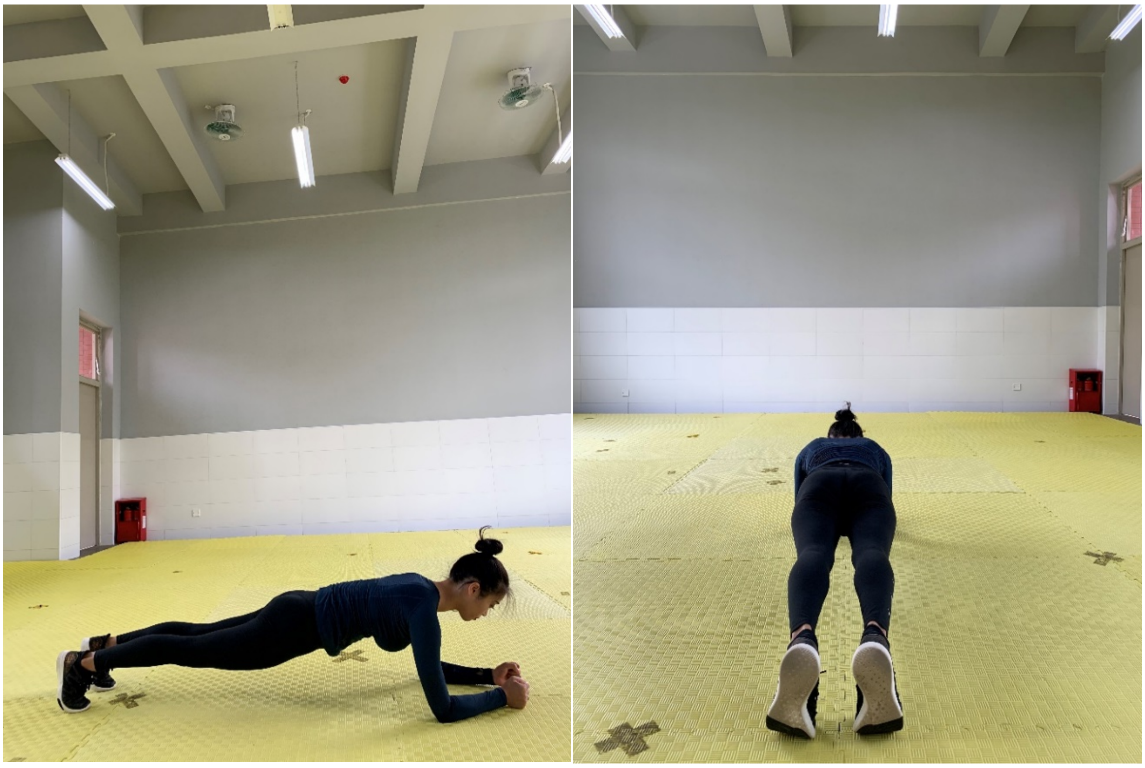

9. One leg hip bridge (bottom point 0.43 × 2)

Left leg support:

- Lie on your back with your legs shoulder-width apart and your hands at your sides;
- Bend your knees with your heels near your hips;
- Keep your left calf perpendicular to the floor and your top hip tucked in so that your torso is in a plane with your left thigh;
- Keep your right leg suspended with your knees bent 90°;
- Keep your left foot on the floor;
- Support your waist, abdomen and lower limbs.

Right leg support:

- Ditto

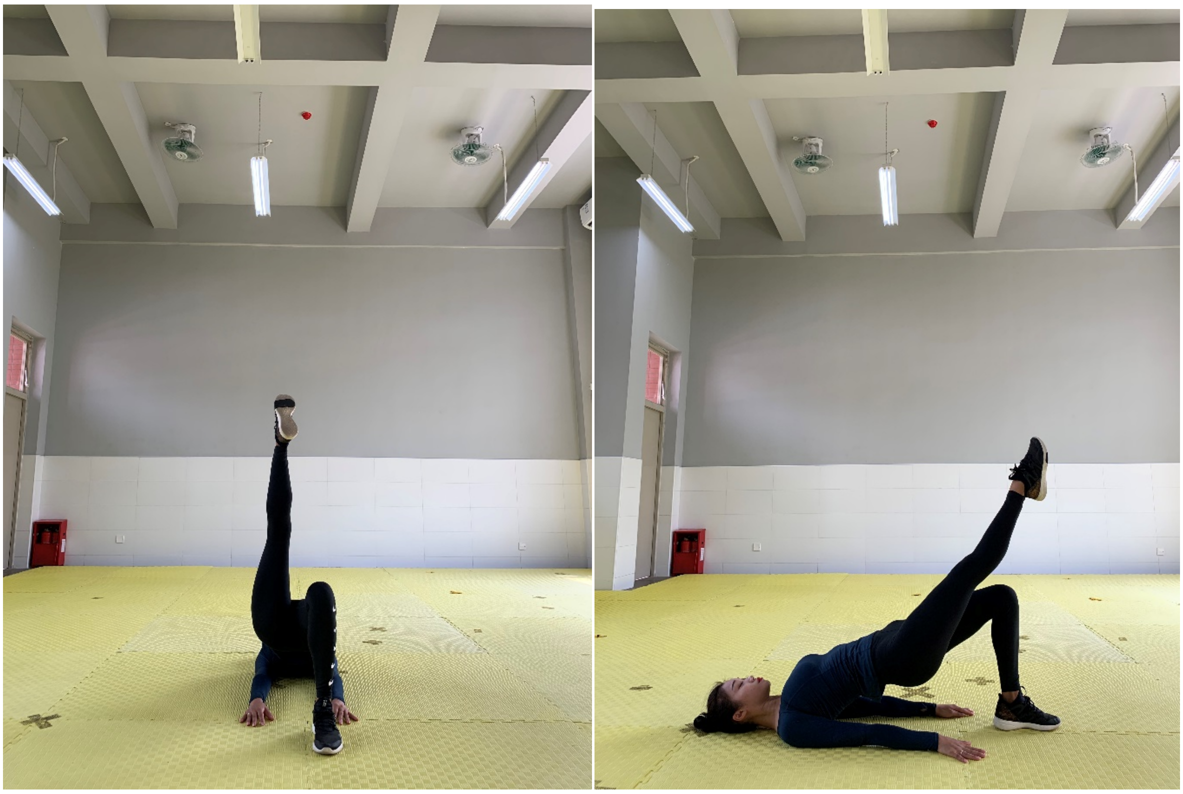

10. Reverse two end start (base point 0.06)

- Starting position: Lie on your stomach with your hands above your head and extended slightly wider than shoulder width;
- The upper limbs and lower limbs rise and fall at the same time;
- Lift above the chest and below the knees while leaving the ground;
- Lower back power control.

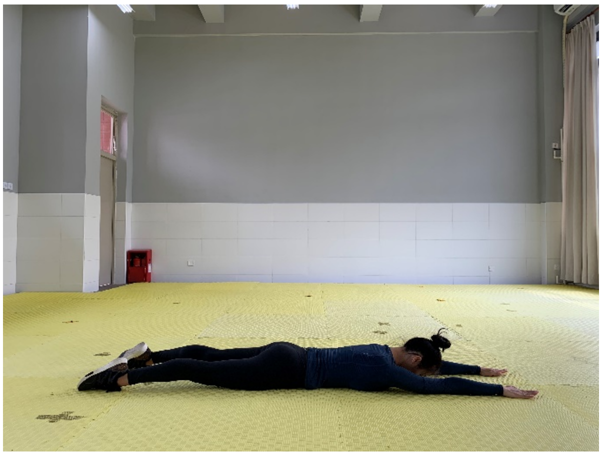

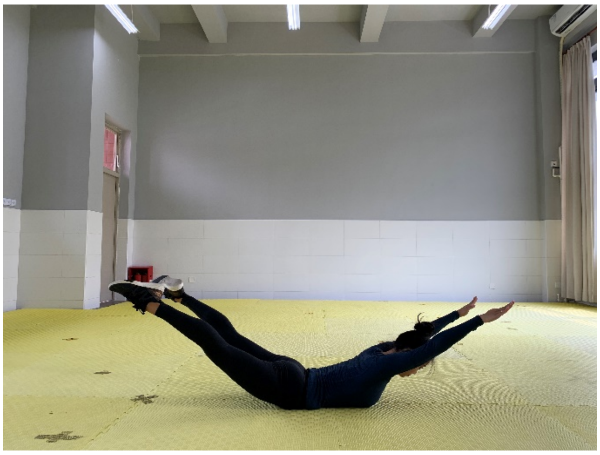

二、 Intervention part (self-weight Squats) :

A. Assisted Squat (12 times with 0.27 × 2; 10 times with 0.3 × 2; 8 times bottom part is about 0.42 × 2)

- Stand upright with feet slightly wider than shoulder width apart, extend arms diagonally upward and put hands on auxiliary stabilizing objects;
- Bend the hips, bend the knees and squat until the thigh is close to the calf and stand up.
- The knee joint does not exceed the toe;
- Knee joint and feet in the same direction forward;
- When standing up, press down on the anchor with both hands to help exert force.

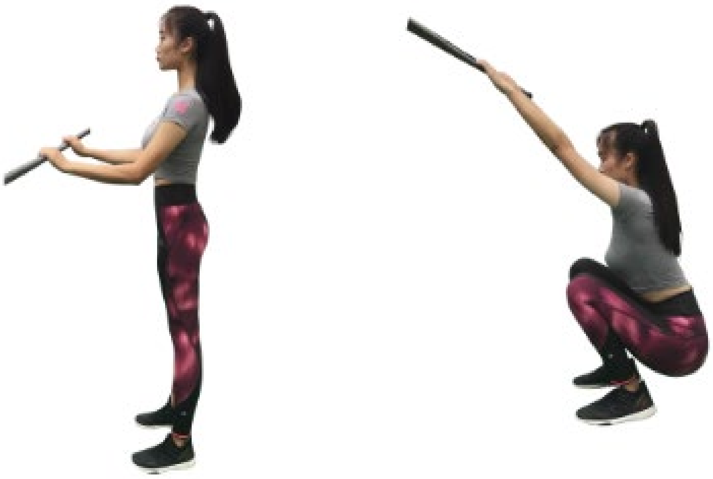

B. Squat (with about 0.32 × 2 on the bottom; 10 scatters of × 0.4 × 2; 1 × 8 × 2)

- Body upright, feet slightly wider than shoulder width apart, arms straight before flat lift;
- Squat with hip flexion and knees bent until the thigh is parallel to the ground and then stand up

and restore;

- Do not exceed the toe of the knee joint;
- The knee joint is facing forward in the same direction as the foot.

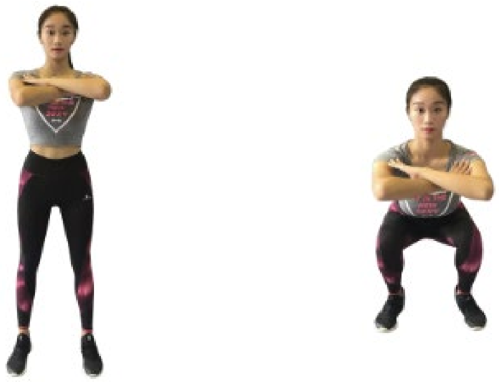

C. Narrow squat (with 12 times of 1 × 2; 10 times with 0.4 × 2; 1 × 8 × 2)

- Body upright, feet together to keep all feet on the ground, arms straight before the flat lift;
- Bend the hips and bend the knees and squat until the back of the thigh is close to the calf and then stand up and restore;
- The front end of the knee does not exceed the tip of the foot;
- The knee joint faces forward in the same direction as the feet.

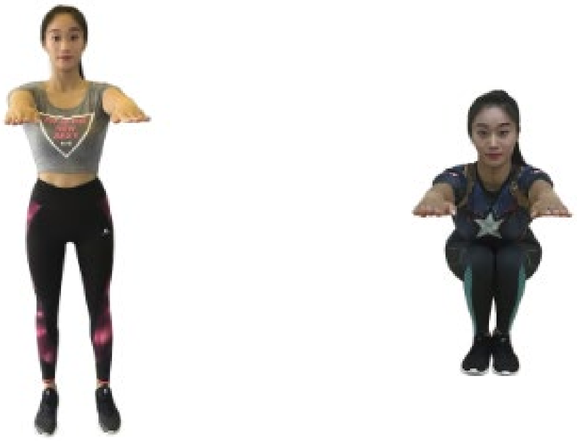

D. Side squat (with 12 repetitions of a 0.14 × 2 bottom; (10 times with 0.17 × 2; 8 times bottom part is about 0.21 × 2)

Left side:

- Keep your head up, waist and back straight, feet open, about twice shoulder width, right leg straight and angled outward 60 degrees, hands hanging at the side of the body;

- Left leg hip flexion squat, until the thigh is parallel to the ground after standing up to restore;
- Do not exceed the toe of the knee;
- Knee joint and feet keep the same direction forward;
- Squat and stand up when the left leg mainly exert force.

Right side:

- Ditto

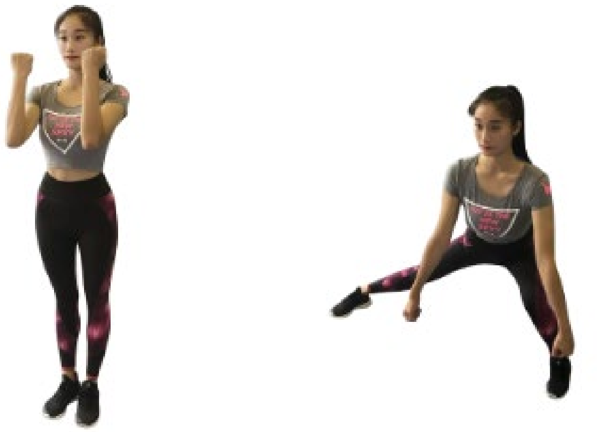

E. Lung (12 times bottom point 0.12 × 2; 0.14 × 2 in 10 times; 8 × 0.18 × 2)

Left side:

- Keep your head up, waist and back straight, torso perpendicular to the ground, hands akimbo on the side of the body, legs apart, shoulder width apart;
- Step forward with your front left leg and bend your knees to squat until your thigh is parallel to the floor;
- Bend your right knee 90 degrees at the back knee with your lower leg parallel to the floor;
- The left knee joint should not exceed the tip of the foot;
- Knee joint and feet keep the same direction forward;
- Buttocks of both legs and legs back to starting standing position.

Right side:

- Ditto

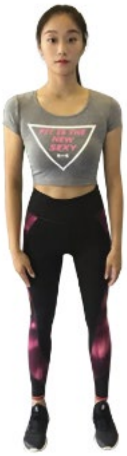

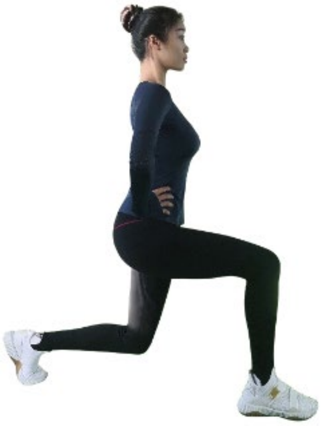

F. **Bulgarian single leg squat (about 0.12 × 2; 10 times = 0.14 × 2; 8 times bottom part of × 0.18 2)**

Left side:

- High score leg posture, left leg in front, right leg behind, right foot instep on the bench, keep the chest straight back, abdomen tightening, hands akimbo, body center of gravity always keep in the neutral position;
- Bend your knees, drop your body into a low-point leg squat position, so that your right knee is close to the ground (about one punch height from the ground);
- Leave knee at a 90° Angle with thigh parallel to the floor;
- The knee joint of the left leg should not exceed the tip of the foot;
- Knee joint and feet keep the same direction forward;
- Push the left hip and leg to stand up.

Right side:

- Ditto

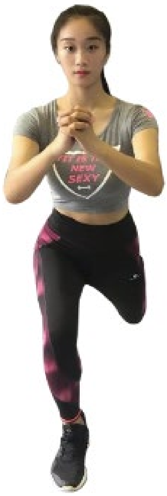

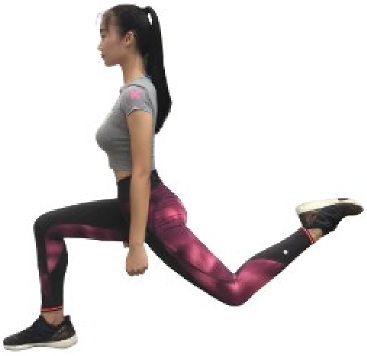

G. Skating squat (12 times with a 1 × 2 bottom; 10 × 10 × 0.125 × 2; 8 × × 0.15625 2)

Left side:

- Keep your head up, eyes ahead, back straight, standing with your left leg supported and right foot slightly lifted off the ground;
- Squat with your left leg bent and your right leg bent backward (similar to kneeling on one knee);
- Squat with your upper body slightly forward and holding steady;
- Squat your left leg to 120 degrees before lifting it;
- Support leg hip, knee, ankle joint always in a straight line;
- Center of gravity as for the left foot sole;
- The right foot does not touch the ground.

Right side:

- Ditto

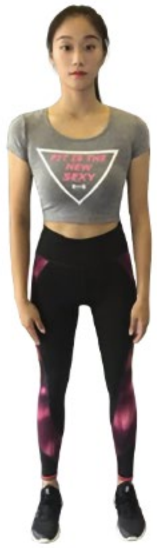

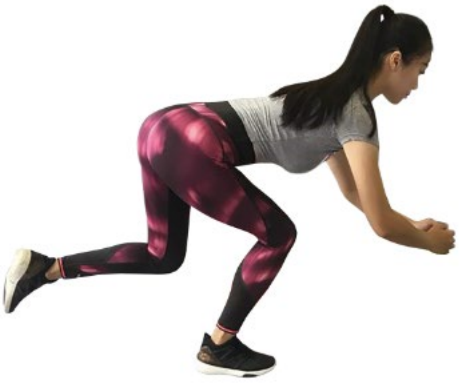

H. Single leg squat (120°) (12 times with about 0.14 × 2; 10 × 1 × 0.17 × 2; 8 times bottom part is about 0.21 × 2)

Left side:

- The body is upright, the waist and back are straight, the arms are straight before the flat lift, the left leg is upright support, the right leg is straight forward and raised, while the right foot is the same height as the left leg knee;
- Bend the hip with the left leg until the knee is close to 90 degrees and then push yourself back to the starting position;
- Keep your right leg in the air;

- The knee joint of the left leg should not exceed the tip of the foot;
- The knee joint is facing forward in the same direction as the feet.

Right side:

- Ditto

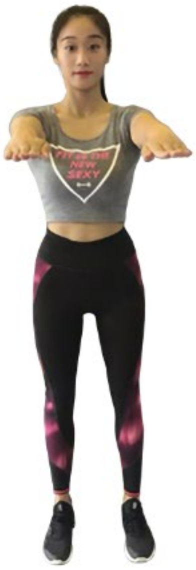

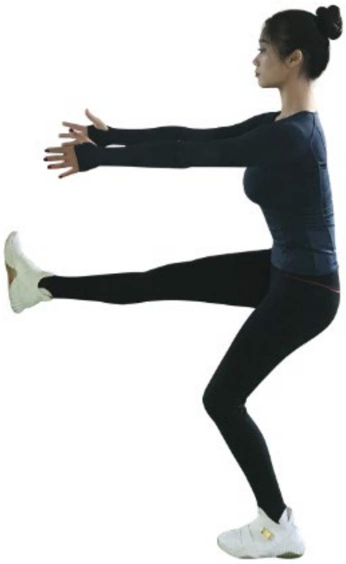

I. Bench pistol squat (12 bottom points about 0.14 × 2; 10 times bench pistol squat (about 0.17 × 2; 8 times bottom part is about 0.21 × 2)

Left side:

- The body is upright, the waist and back are straight, standing in front of the bench a foot long distance, arms straight before flat lift, left leg upright support, right leg straight forward lift, at the same time the right foot and left leg knee height is consistent;
- Bend the hip and the knee of the left leg, fall and control the force until the buttocks sit on the bench after the force to push themselves back to the starting position;
- The right leg always hangs in the air during squatting;
- The knee joint of the left leg should not exceed the toe;
- The knee joint is facing forward in the same direction as the feet.

Right side:

- Ditto

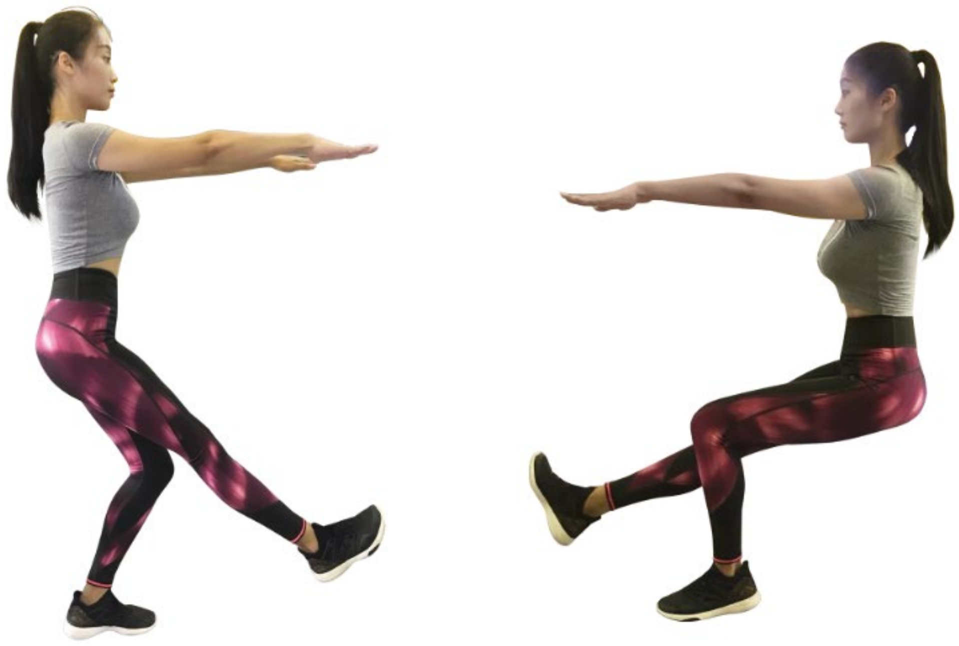

J. Auxiliary pistol squat (12 bottom points about 0.14 × 2; 10 times with 0.17 × 2 bottom points; 8 times bottom part is about 0.21 × 2)

Left side:

- The body is upright, the waist and back are straight, the left arm naturally hangs to the side of the body, the medicine ball is located on the outside of the foot of the left leg, the right arm is raised flat in front, the left leg is supported upright, the right leg is lifted straight forward, and the height of the right foot is consistent with the knee of the left leg;
- The left leg from upright to hip flexion, until the back of the thigh close to the calf, the palm supported on the medicine ball after lifting;
- The knee joint of the left leg should not exceed the toe;
- Knee joint and feet keep the same direction forward;
- Place your left hand on the medicine ball and press the ball as you rise to assist your left leg in lifting back to the initial stage.

Right side:

- Ditto

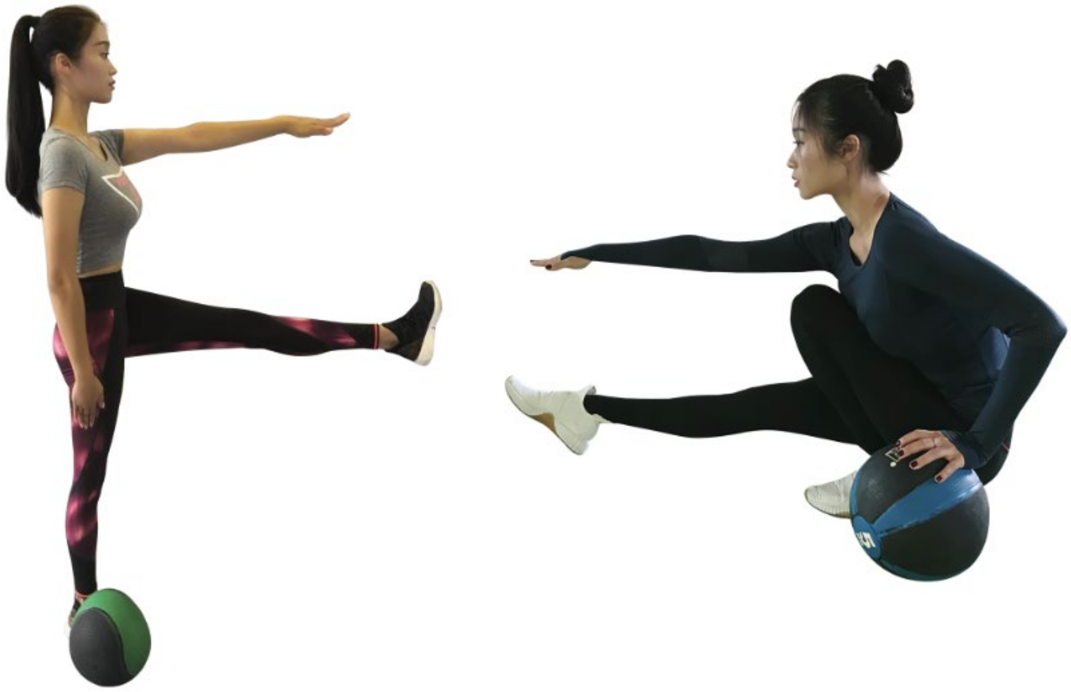

三、 Intervention part (Barbell Squats) :

1、Barbell squat requirements (12 base points about 0.28; 10 bottom points about 0.33; 8 bottoms about 0.42)

- Keep your chest out and head up, your body erect, shoulder blades contracted, and back straight;
- Bend the knees and hips until the thighs are nearly parallel to the ground and the torso is parallel to the tibia
- The knees of both legs should not exceed the feet too much;
- Knee joint and feet keep the same direction forward;
- The sole of the foot touches the floor.

四、 The Stretch part:

1. One leg back stretch (bottom part 0.625 × 2)

Left side

- Straighten your left leg and bend your right leg back, bringing the ball of your right foot to the base of your left thigh;
- Extend your arms up from both sides, palms facing each other;
- Keep your waist and back straight down;
- Place your hands at the farthest end of the floor, with your chest near your legs;
- The left leg is stretched at the back of the thigh and the hamstring level.

Right side:

- Ditto

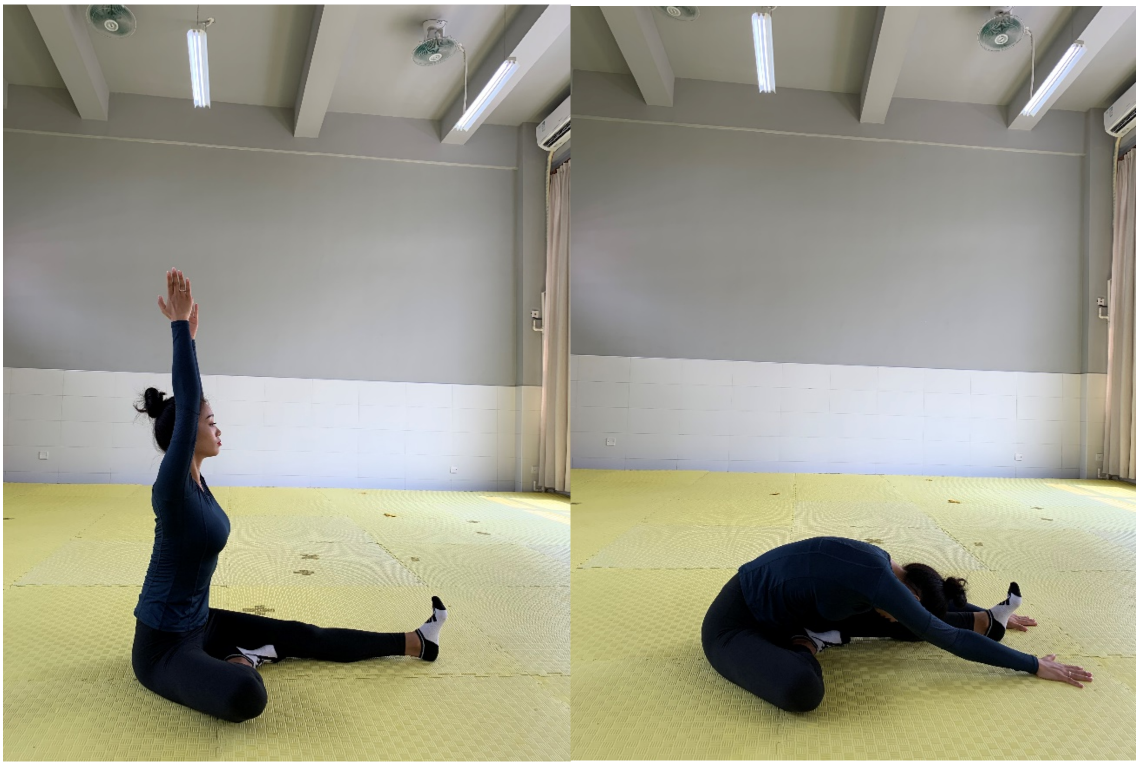

2. Seated Angle pose (bottom part 1.25 × 2)

- Sit on the floor with legs spread to the limit;
- Extend your arms up from both sides, palms facing each other;
- Keep your back straight and your body down;
- Keep your hands in front of you and your body close to the floor;
- Feel a stretch in the hips of both legs, the inner thighs and the inside of the knee joint.

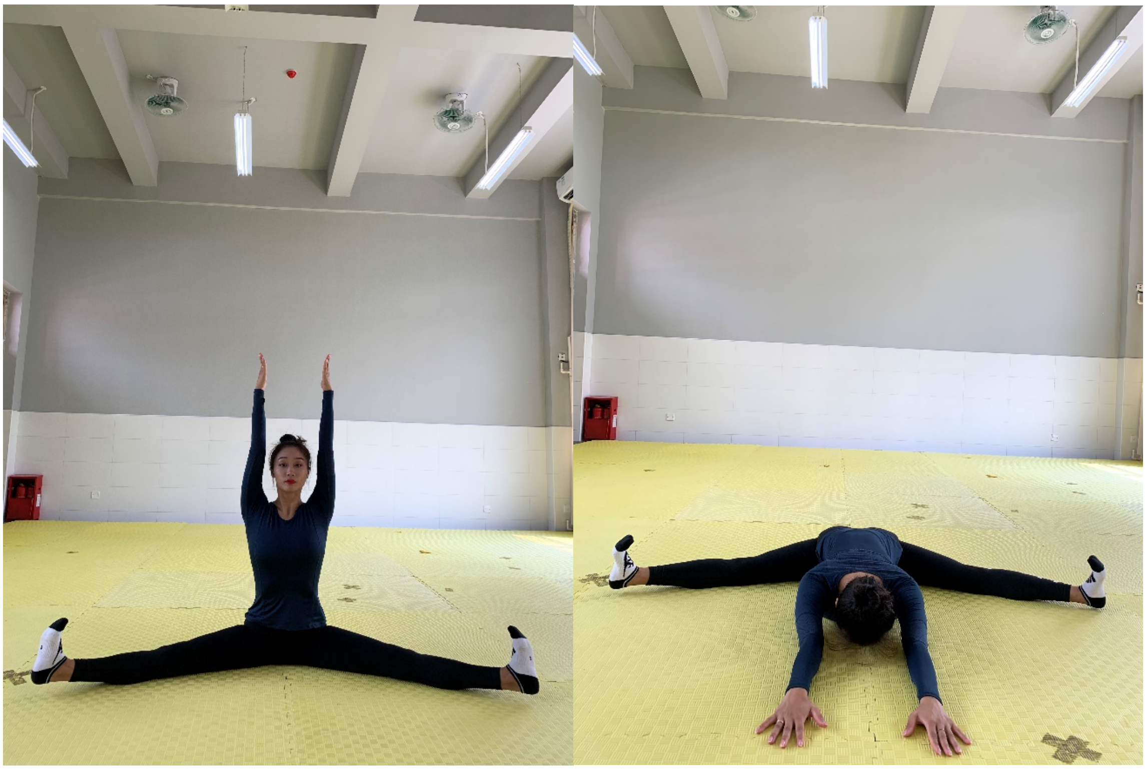

3. Gluteal muscle stretch in sitting posture (0.75 × 2 bottom)

Left side:

- Left leg in front, big calf folded on the ground, bend your knees about 60 degrees, right leg straight behind, arms straight in front of you;
- Keep your torso neutral and down with your chest close to the floor;
- Stretch the right psoas major muscle;
- Stretch the biceps femoris muscle of the leg, the lateral femoris muscle and the hip muscle have a stretching sensation.

Right side:

- Ditto

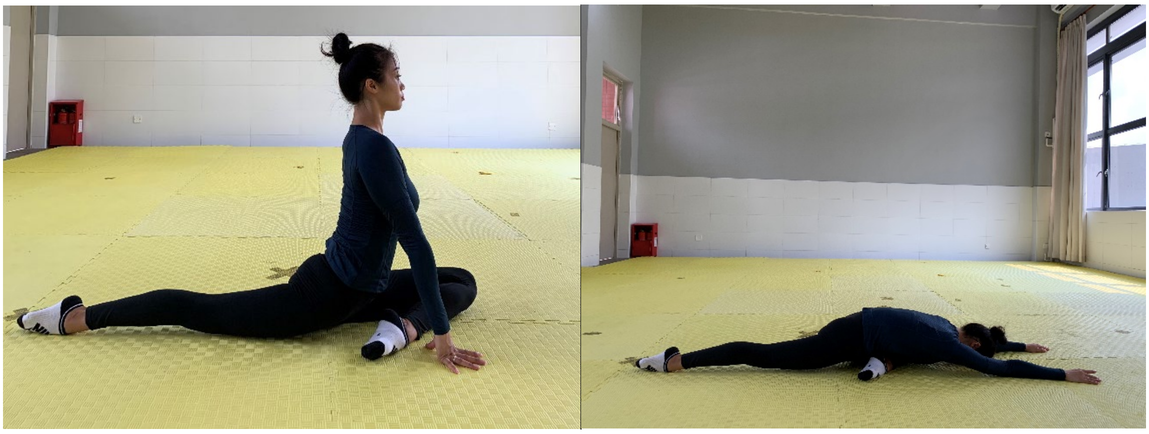

4. Quadriceps stretch (prone) (bottom point 0.9375 × 2)

Left side:

- Lie on your side with your torso still upright and relaxed, with your left leg bent back, your big calf folded, and your right leg straight and relaxed;
- Hold the left ankle in the left hand to pull the left leg back, so that the left thigh quadriceps muscle stretch;
- Breathe naturally during the process, and the Angle between your legs should not exceed 30°.

Right side:

- Ditto

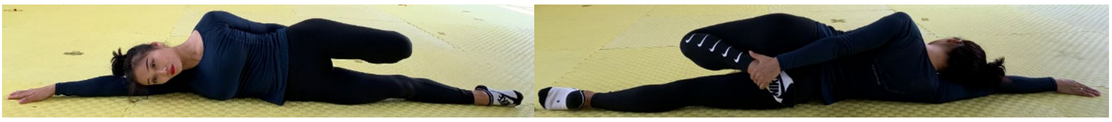

5. Gastrocnemius stretching (bottom part 0.75 × 2)

Left side:

- Both hands and the front palm of the left leg, the right foot placed on the lower end of the calf gastrocnemius stretched leg;
- Hips up, body into an inverted V shape;
- The left leg back heel force active downward pressure;
- Stretching sensation in left leg calf.

Right side:

- Ditto

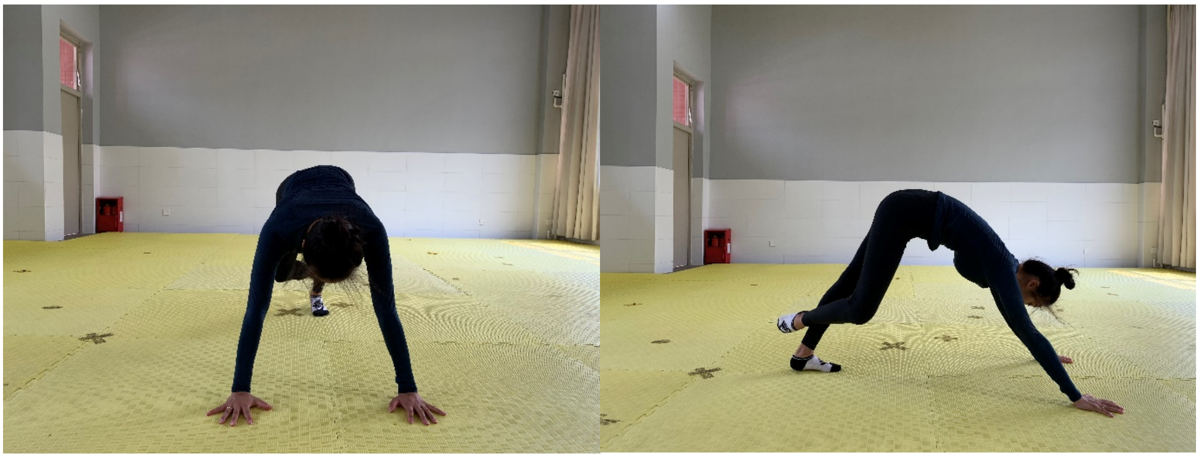

6. The cobra pose (base score 1.875)

- Lie on your stomach with your legs together and straight, hands on either side of your chest, palms down, elbows close to your ribs;
- Lift your body with your arms until they are completely straight;
- The pubic bone touches the floor, while the waist and spine feel stretched.

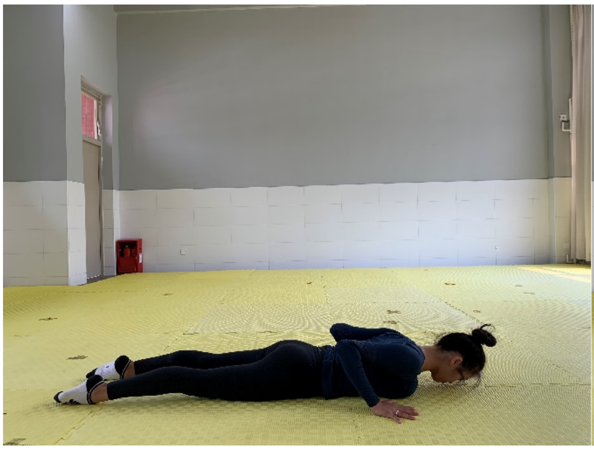
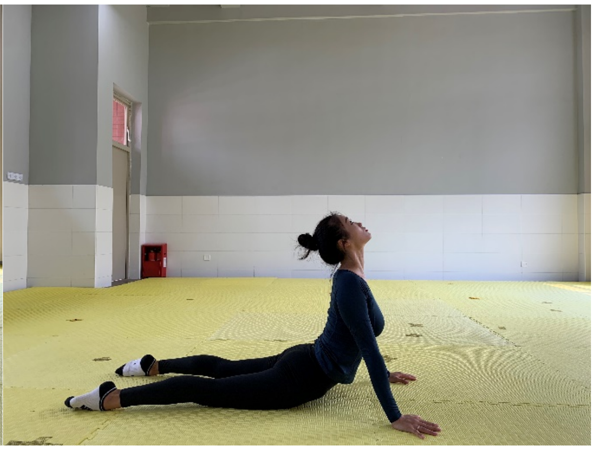

7. Reclined spinal twist (bottom part 0.625 × 2)

- Supine position with arms extended to the sides in a straight line;
- Lift your legs with your knees bent and turn to your left side, keeping your legs together and your left leg against the floor;
- Extend your right leg straight until the tip of your right foot touches the tip of your left hand;
- Stretch the shoulders, chest and lower back;
- Hips and hamstrings.

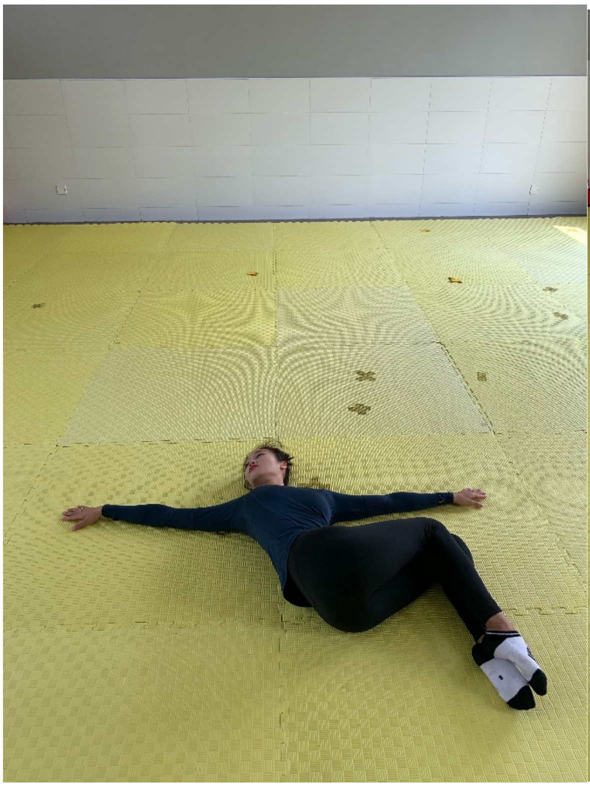
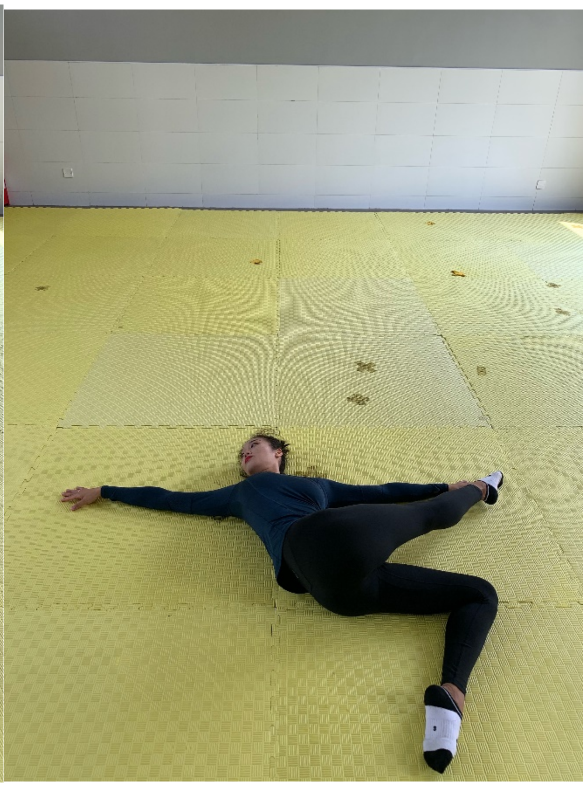

Supplement: Supplementary file 2 — Supplementary Information 2. [file 41598_2023_40319_MOESM2_ESM.pdf]
