# Supplementary material for: Effects of progressive body-weight versus barbell back squat training on strength, hypertrophy and body fat among sedentary young women
Source: Sci Rep. 2023 Aug 19;13:13505. doi: 10.1038/s41598-023-40319-x (PMC10439966; doi:10.1038/s41598-023-40319-x)
Supplement: Supplementary file 3 — Supplementary Tables. [file 41598_2023_40319_MOESM3_ESM.pdf]

Table 1. Bodyweight squat progression (a)

Subjects	First session		Second session		Third session		Fourth session	
	level	R × S	level	R × S	level	R × S	level	R × S
1	E/F/G/G/G/G	12/10/8/8/8/8	E/F/G/G/G/G	12/10/10/10/10/8	E/F/G/G/G/G	12/10/10/10/10/10	E/F/G/G/G/G	12/10/10/10/10/10
2	E/F/G/G/G/G	12/10/8/8/8/8	E/F/G/G/G/G	12/10/10/10/8/8	E/F/G/G/G/G	12/10/10/10//10	E/F/G/G/G/G	12/10/10/10/10/10
3	E/F/G/G/G/G	12/10/8/8/8/8	E/F/G/G/G/G	12/10/10/10/10/8	E/F/G/G/G/G	12/10/10/10/10/10	E/F/G/G/G/G	12/10/10/10/10/10
4	E/F/G/G/G/G	12/10/8/8/8/8	E/F/G/G/G/G	12/10/10/10/10/8	E/F/G/G/G/G	12/10/10/10/10/10	E/F/G/G/G/G	12/10/10/10/10/10
5	E/F/G/G/G/G	12/10/8/8/8/8	E/F/G/G/G/G	12/10/10/10/10/8	E/F/G/G/G/G	12/10/10/10/10/10	E/F/G/G/G/G	12/10/10/10/10/10
6	E/F/G/G/G/G	12/10/8/8/8/8	E/F/G/G/G/G	12/10/10/8/8/8	E/F/G/G/G/G	12/10/10/10/10/10	E/F/G/G/G/G	12/10/10/10/10/10

Table 1. Bodyweight squat progression (b)

Subjects	Fifth session		Sixth session		Seventh session		Eighth session	
	level	R × S	level	R × S	level	R × S	level	R × S
1	E/F/H/G/G/G	12/10/6/10/10/10	F/G/H/H/H/H	12/10/8/8/8/8	F/G/H/H/H/H	12/10/8/8/8/8	F/G/H/H/H/H	12/10/10/10/10/10
2	F/G/H/H/H/H	12/10/8/8/8/8	F/G/H/H/H/H	12/10/10/8/8/8	F/G/H/H/H/H	12/10/10/10/10/10	G/H/I/I/I/I	12/10/8/8/8/8
3	F/G/H/H/H/H	12/10/8/8/8/8	F/G/H/H/H/H	12/10/10/8/8/8	F/G/H/H/H/H	12/10/10/10/10/10	G/H/I/I/I/I	12/10/8/8/8/8
4	F/G/H/H/H/H	12/10/8/8/8/8	F/G/H/H/H/H	12/10/10/10/8/8	F/G/H/H/H/H	12/10/10/10/10/10	G/H/I/I/I/I	12/10/8/8/8/8
5	E/F/H/H/G/G	12/10/6/6/10/10	F/G/H/H/H/H	12/10/8/8/8/8	F/G/H/H/H/H	12/10/10/10/8/8	F/G/H/H/H/H	12/10/10/10/10/10
6	E/F/H/G/G/G	12/10/6/10/10/10	F/G/H/H/H/H	12/10/8/8/8/8	F/G/H/H/H/H	12/10/10/8/8/8	F/G/H/H/H/H	12/10/10/10/10/10

Table 1. Bodyweight squat progression (c)

Subjects	Ninth session		Tenth session		Eleventh session		Twelfth session	
	level	R × S	level	R × S	level	R × S	level	R × S
1	F/G/I/G/G/G	12/10/6/10/10/10	F/G/I/I/G/G	12/10/8/8/10/10	G/H/I/I/I/I	12/10/8/8/8/8	G/H/I/I/I/I	12/10/10/10/10/8
2	G/H/I/I/I/I	12/10/10/10/8/8	G/H/I/I/I/I	12/10/10/10/10/10	G/H/J/I/I/I	12/10/6/10/10/10	G/H/J/J/J/I	12/10/8/8/6/10
3	G/H/I/I/I/I	12/10/10/10/10/8	G/H/I/I/I/I	12/10/10/10/10/10	G/H/J/I/I/I	12/10/6/10/10/10	G/H/J/J/J/J	12/10/8/8/8/6
4	G/H/I/I/I/I	12/10/10/8/8/8	G/H/I/I/I/I	12/10/10/10/10/8	G/H/I/I/I/I	12/10/10/10/10/10	G/H/J/J/I/I	12/10/8/6/10/10
5	G/H/I/I/I/I	12/10/8/8/8/8	G/H/I/I/I/I	12/10/10/10/10/8	G/H/I/I/I/I	12/10/10/10/10/10	G/H/J/I/I/I	12/10/6/10/10/10
6	E/F/I/I/I/I	12/10/8/8/8/8	G/H/I/I/I/I	12/10/10/10/8/8	G/H/I/I/I/I	12/10/10/10/10/10	G/H/J/I/I/I	12/10/6/10/10/10

Note: Repetitions × Sets = R × S; 1-6 represents each participant; E: Lunge, F: Bulgarian single leg squat, G: Skating squat; The table shows the training volume and movement level (A -J) completed by the 6 participants in the bodyweight squat group in the first four training sessions.

Table 2. Barbell back squat progression (a)

Subjects	First session		Second session		Third session		Fourth session	
	weight	R × S	weight	R × S	weight	R × S	weight	R × S
1	30/32/34/34/34/34	12/10/9/9/8/8	30/32/34/34/34/34	12/10/9/9/9/9	30/32/34/34/34/34	12/10/10/10/10/10	30/32/34/34/34/34	12/10/10/10/10/10
2	40/44/46/46/46/46	12/10/9/9/8/8	40/44/46/46/46/46	12/10/9/9/9/9	40/44/46/46/46/46	12/10/10/10/10/10	40/44/46/46/46/46	12/10/10/10/10/10
3	40/44/46/46/46/46	12/10/9/8/8/8	40/44/46/46/46/46	12/10/9/9/9/9	40/44/46/46/46/46	12/10/10/10/10/10	40/44/46/46/46/46	12/10/10/10/10/10
4	46/50/54/54/54/54	12/10/9/8/8/8	46/50/54/54/54/54	12/10/9/9/9/9	46/50/54/54/54/54	12/10/10/10/10/10	46/50/54/54/54/54	12/10/10/10/10/10
5	40/44/46/46/46/46	12/10/9/9/8/8	40/44/46/46/46/46	12/10/9/9/9/9	40/44/46/46/46/46	12/10/10/10/10/10	40/44/46/46/46/46	12/10/10/10/10/10
6	40/44/46/46/46/46	12/10/9/9/8/8	40/44/46/46/46/46	12/10/9/9/9/9	40/44/46/46/46/46	12/10/10/10/10/10	40/44/46/46/46/46	12/10/10/10/10/10
7	50/56/60/60/60/60	12/10/9/8/8/8	50/56/60/60/60/60	12/10/9/9/9/9	50/56/60/60/60/60	12/10/10/10/10/10	50/56/60/60/60/60	12/10/10/10/10/10

Table 2. Barbell back squat progression (b)

Subjects	First session		Second session		Third session		Fourth session	
	weight	R × S	weight	R × S	weight	R × S	weight	R × S
1	32.5/34.5/36.5/36.5/36.5/36.5	12/10/8/8/8/8	32.5/34.5/36.5/36.5/36.5/36.5	12/10/9/9/9/9	32.5/34.5/36.5/36.5/36.5/36.5	12/10/10/10/10/10	35/37/39/39/39/39	12/10/8/8/7/6
2	42.5/46.5/48.5/48.5/48.5/48.5	12/10/8/8/8/8	42.5/46.5/48.5/48.5/48.5/48.5	12/10/9/9/9/9	42.5/46.5/48.5/48.5/48.5/48.5	12/10/10/10/10/10	45/49/51/48.5/48.5/48.5	12/10/5/10/7/7
3	42.5/46.5/48.5/48.5/48.5/48.5	12/10/8/8/8/8	42.5/46.5/48.5/48.5/48.5/48.5	12/10/9/9/9/9	42.5/46.5/48.5/48.5/48.5/48.5	12/10/10/10/10/10	46.5/48.5/51/51/48.5/48.5	12/10/8/7/8/8
4	48.5/52.5/56.5/56.5/56.5/56.5	12/10/8/8/8/8	48.5/52.5/56.5/56.5/56.5/56.5	12/10/9/9/9/9	48.5/52.5/56.5/56.5/56.5/56.5	12/10/10/10/10/10	51/55/59/59/59/56.5	12/10/8/8/6/8
5	42.5/46.5/48.5/48.5/48.5/48.5	12/10/8/8/8/8	42.5/46.5/48.5/48.5/48.5/48.5	12/10/9/9/9/9	42.5/46.5/48.5/48.5/48.5/48.5	12/10/10/10/10/10	42.5/46.5/48.5/48.5/48.5/48.5	12/10/10/10/10/9
6	42.5/46.5/48.5/48.5/48.5/48.5	12/10/8/8/8/8	42.5/46.5/48.5/48.5/48.5/48.5	12/10/9/9/9/9	42.5/46.5/48.5/48.5/48.5/48.5	12/10/10/10/10/10	45/49/51/51/51/51	12/10/8/8/8/8
7	52.5/58.5/62.5/62.5/62.5/62.5	12/10/8/8/8/8	52.5/58.5/62.5/62.5/62.5/62.5	12/10/9/9/9/9	52.5/58.5/62.5/62.5/62.5/62.5	12/10/10/10/10/10	55/61/65/62.5/62.5/62.5	12/10/6/10/10/10

Table 2. Barbell back squat progression (c)

Subjects	First session		Second session		Third session		Fourth session	
	weight	R × S	weight	R × S	weight	R × S	weight	R × S
1	35/37/39/39/39/39	12/10/9/8/8/8	35/37/39/39/39/39	12/10/10/10/10/10	37.5/39.5/41.5/41.5/41.5/41.5	12/10/8/8/8/6	37.5/39.5/41.5/41.5/41.5/41.5	12/10/10/10/9/8
2	45/49/51/48.5/48.5/48.5	12/10/6/7/7/5	45/49/51/51/51/51	12/10/8/8/8/6	45/49/51/51/51/51	12/10/9/9/8/8	45/49/51/51/51/51	12/10/8/10/10/10
3	46.5/48.5/51/51/51/51	12/10/10/8/8/8	46.5/48.5/51/51/51/51	12/10/10/10/10/10	49/51/53.5/53.5/51/51	12/10/8/7/10/9	49/51/53.5/53.5/53.5/53.5	12/10/8/8/8/6

4	51/55/59/59/59/59	12/10/8/8/8/8	51/55/59/59/59/59	12/10/10/10/10/8	51/55/59/59/59/59	12/10/10/10/10/10	53.5/57.5/61.5/61.5/61.5/61.5	12/10/8/8/8/7
5	42.5/46.5/48.5/48.5/48.5/48.5	12/10/10/10/10/10	45/49/51/51/51/48.5	12/10/8/8/7/9	45/49/51/51/51/51	12/10/8/8/8/8	45/49/51/51/51/51	12/10/9/9/8/9
6	45/49/51/51/51/51	12/10/8/8/8/7	45/49/51/51/51/51	12/10/9/9/8/8	45/49/51/51/51/51	12/10/10/10/10/8	45/49/51/51/51/51	12/10/10/10/10/10
7	55/61/65/65/65/62.5	12/10/8/8/6/10	55/61/65/65/65/65	12/10/9/10/8/8	55/61/65/65/65/65	12/10/10/10/10/8	55/61/65/65/65/65	12/10/10/10/10/10
